# MSC-EVs in Cartilage Regeneration and Immunomodulation: Mechanisms and Therapeutic Prospects for Osteoarthritis, Rheumatoid Arthritis and Intervertebral Disc Degeneration

**DOI:** 10.3390/ijms27188208

**Published:** 2026-09-15

**Authors:** Tong Ming Liu

**Affiliations:** Institute of Molecular and Cell Biology (IMCB), Agency for Science, Technology and Research (A*STAR), 61 Biopolis Drive, Singapore 138673, Singapore; dbsliutm@yahoo.com

**Keywords:** MSC-EV, osteoarthritis, rheumatoid arthritis, intervertebral disc degeneration, cartilage repair, inflammation modulation

## Abstract

Mesenchymal stem cell-derived extracellular vesicles (MSC-EVs) represent a promising cell-free therapeutic strategy for cartilage regeneration and inflammation modulation in degenerative and inflammatory musculoskeletal disorders, including osteoarthritis (OA), rheumatoid arthritis (RA) and intervertebral disc degeneration (IVDD). MSC-EVs enhance cartilage repair by promoting chondrocyte proliferation, migration, survival and extracellular matrix (ECM) synthesis to maintain cartilage homeostasis. In parallel, they exert anti-inflammatory and anti-catabolic effects by suppressing inflammatory cytokines, matrix-degrading enzymes, oxidative stress and inflammasome activation. In OA, MSC-EVs regulate chondrocyte function, ECM remodelling, immune responses and tissue regeneration by modulating multiple signalling pathways, including NF-κB, PI3K/AKT, MAPK, Wnt/β-catenin and YAP signalling. In RA, MSC-EVs orchestrate innate and adaptive immune regulation, metabolic reprogramming and tissue repair pathways. In IVDD, MSC-EVs suppress chronic inflammation, reduce nucleus pulposus cell apoptosis, enhance cell proliferation and promote ECM synthesis, facilitating disc regeneration and functional restoration. Despite their therapeutic potential, several challenges hinder clinical translation. This review summarises current understanding of MSC-EV-mediated mechanisms in OA, RA and IVDD, and proposes strategies to enhance therapeutic efficacy and accelerate clinical application in musculoskeletal regenerative medicine.

## 1. Introduction

Mesenchymal stem cells (MSCs) are among the most extensively investigated stem cell populations in regenerative medicine, with more than 1500 registered clinical trials evaluating their therapeutic potential across over 30 diseases. Despite encouraging preclinical and clinical outcomes, the widespread clinical translation of MSC-based therapies remains limited by several challenges, including donor-to-donor heterogeneity, poor cell survival and engraftment after transplantation, immune rejection, pulmonary capillary entrapment following systemic administration, potential tumorigenicity and the complexity of manufacturing and regulating living cell products. Increasing evidence indicates that the therapeutic benefits of MSCs are mediated primarily through paracrine mechanisms rather than direct tissue engraftment, with extracellular vesicles (EVs) recognised as key mediators of these effects.

MSC-derived extracellular vesicles (MSC-EVs) are nanosized lipid bilayer-enclosed vesicles that transport a diverse repertoire of bioactive molecules, including proteins, lipids, metabolites, cytokines, mRNAs, microRNAs (miRNAs), long non-coding RNAs (lncRNAs), circular RNAs (circRNAs) and other regulatory nucleic acids [1,2]. By delivering these cargos to recipient cells, MSC-EVs regulate cell proliferation, survival, migration, angiogenesis, extracellular matrix (ECM) remodelling and immune responses. Compared with parental MSCs, MSC-EVs offer several advantages, including lower immunogenicity, improved biosafety, negligible risk of tumour formation or vascular occlusion, enhanced stability during storage and transportation, greater tissue penetration and compatibility with scalable manufacturing and bioengineering strategies. These characteristics have established MSC-EVs as one of the most promising cell-free therapeutic platforms for regenerative medicine [2,3].

Accumulating evidence indicates that MSC-EVs are highly heterogeneous, and their biological activities are largely determined by the tissue origin and physiological status of the parental MSCs. EVs derived from bone marrow, adipose tissue, umbilical cord, synovium, gingiva and induced pluripotent stem cell-derived MSCs (iMSCs) possess distinct molecular cargos and functional properties, resulting in marked differences in their regenerative, chondroprotective, angiogenic and immunomodulatory capacities [4]. Among these sources, bone marrow-derived mesenchymal stromal cells (BMSCs) and adipose-derived mesenchymal stromal cells (ADSCs) are among the most extensively studied MSC-EV sources, particularly in regenerative medicine and musculoskeletal applications. Although EVs from both cell types exhibit anti-inflammatory, pro-regenerative and immunomodulatory activities, increasing evidence indicates that their therapeutic efficacy differs according to the tissue origin of the parental MSCs. BMSC-EVs are generally considered to possess stronger immunomodulatory and osteochondral regenerative properties [5,6]. They are enriched in immunoregulatory proteins and microRNAs that suppress pro-inflammatory cytokine production, promote macrophage polarisation toward the anti-inflammatory M2 phenotype, inhibit T cell activation and enhance cartilage and bone regeneration. Consequently, BMSC-EVs have been widely investigated for the treatment of osteoarthritis (OA), rheumatoid arthritis (RA), graft-versus-host disease (GVHD), bone defects and other immune-mediated disorders.

In contrast, ADSC-EVs are more closely associated with angiogenesis, wound healing and soft tissue regeneration. Owing to the unique secretory profile of adipose-derived MSCs, ADSC-EVs are enriched in pro-angiogenic growth factors and regenerative microRNAs that enhance endothelial cell proliferation and migration, inhibit apoptosis, accelerate cutaneous wound healing and improve tissue vascularization. As a result, ADSC-EVs have demonstrated considerable therapeutic potential in skin repair, cardiovascular diseases, diabetic wound healing, peripheral nerve injury, and cosmetic and reconstructive medicine [7,8]. Comparative studies further demonstrate that ADSC-EVs outperform BMSC- and synovium-derived MSC-EVs in promoting MSC migration, proliferation, chondrogenic differentiation and cartilage regeneration. These functional differences are associated with distinct proteomic and transcriptomic cargo profiles that regulate signalling pathways involved in focal adhesion, extracellular matrix (ECM)–receptor interaction, actin cytoskeleton organisation and PI3K-Akt signalling [9].

Beyond BMSCs and ADSCs, EVs derived from other MSC sources have also demonstrated unique therapeutic advantages. Umbilical cord-derived MSC-EVs (UC-MSC-EVs) exhibit high production efficiency, whereas both UC-MSC-EVs and dental pulp stem cell-derived EVs (DPSC-EVs) have demonstrated favourable drug-loading and delivery capacities [4]. In a mouse model of osteoarthritis (OA), induced pluripotent stem cell-derived mesenchymal stem cells EVs (iMSC-EVs) exhibit greater efficacy in attenuating disease progression than synovial MSC-derived EVs (SMSC-EVs) [10]. Likewise, gingiva-derived MSC-EVs (GMSC) display potent immunomodulatory activity in rheumatoid arthritis (RA) [11], while iMSC-EVs exhibit enhanced anti-senescence and regenerative effects in intervertebral disc degeneration (IVDD) by delivering miR-105-5p to activate the Sirt6 signalling pathway [12]. These findings highlight that the therapeutic properties of MSC-EVs are source-dependent and underscore the importance of selecting the most appropriate MSC source for disease-specific applications.

In addition to tissue origin, the developmental stage of the parental MSCs is another critical determinant of EV therapeutic efficacy. EVs derived from foetal MSCs (fMSCs) exhibit superior regenerative capacity compared with those derived from perinatal MSCs [13]. Similarly, donor age significantly influences EV function. EVs from young BMSCs more effectively attenuate cellular senescence and preserve cartilage integrity than EVs from aged BMSCs by activating phosphatase and tensin homologue (*PTEN*) and suppressing phosphoinositide 3-kinase/protein kinase B (PI3K/AKT) signalling. In a mouse model of osteoarthritis, EVs derived from young donors significantly delay disease progression, highlighting donor age as an important determinant of the therapeutic efficacy of BMSC-EVs [14].

Furthermore, the biological potency of MSC-EVs is influenced by multiple factors, including donor age, disease status, culture conditions and priming strategies such as hypoxia and inflammatory stimulation. These factors alter the molecular composition of EVs and consequently modulate their regenerative and immunomodulatory functions. Therefore, comprehensive functional characterisation based on molecular cargo and biological activity has become increasingly important for evaluating MSC-EVs, complementing conventional characterisation based on particle size, morphology and surface marker expression. Such multidimensional characterisation will facilitate the rational selection and optimisation of MSC-EVs for disease-specific therapeutic applications.

Physicochemical characterisation is essential for confirming the identity, purity and quality of MSC-EVs. According to the Minimal Information for Studies of Extracellular Vesicles (MISEV) guidelines, characterisation should include particle size and concentration, morphology, EV marker expression and assessment of sample purity. Particle size and concentration are commonly measured by nanoparticle tracking analysis (NTA), nano-flow cytometry (NanoFCM) or tunable resistive pulse sensing (TRPS), while morphology is evaluated by transmission electron microscopy (TEM) or cryo-EM. Canonical EV markers (CD9, CD63, CD81, TSG101, and ALIX) and negative markers (calnexin and GM130) are typically analysed by Western blotting or flow cytometry to verify EV identity and purity [15].

Beyond conventional physicochemical characterisation, several emerging approaches have improved the evaluation of MSC-EV properties. Single-EV analysis techniques, such as NanoFCM, ExoView, and super-resolution microscopy, reveal EV heterogeneity at the individual vesicle level. Multi-omics profiling, including proteomics, small RNA sequencing, lipidomics and metabolomics, provides comprehensive insights into EV cargo and biological functions. Functional potency assays directly assess therapeutic activities, such as immunomodulation, angiogenesis and tissue regeneration, serving as clinically relevant quality attributes. In addition, artificial intelligence (AI)-assisted analysis and high-dimensional single-vesicle profiling integrate complex datasets to identify critical quality attributes, predict therapeutic potency and improve MSC-EV characterisation and manufacturing.

Musculoskeletal disorders, including cartilage injury, osteoarthritis (OA), rheumatoid arthritis (RA) and intervertebral disc degeneration (IVDD), are major causes of chronic pain, disability and reduced quality of life worldwide. Because articular cartilage is avascular, aneural and alymphatic, it possesses very limited intrinsic regenerative capacity [16,17]. Progressive inflammation further accelerates cartilage degeneration by promoting ECM degradation, oxidative stress, cellular senescence and apoptosis. In OA and IVDD, chronic low-grade inflammation disrupts tissue homeostasis and impairs regeneration, whereas persistent autoimmune inflammation in RA drives synovial hyperplasia, immune cell infiltration, cartilage destruction and bone erosion through excessive production of pro-inflammatory cytokines, including TNF-α, IL-1β and IL-6. Consequently, therapeutic strategies capable of simultaneously suppressing inflammation and promoting tissue regeneration are highly desirable.

MSC-EVs have emerged as promising therapeutic candidates for cartilage repair and musculoskeletal regeneration owing to their combined regenerative and immunomodulatory properties [2,3,18]. Numerous preclinical studies have demonstrated that MSC-EVs promote chondrocyte proliferation, enhance ECM synthesis, inhibit apoptosis, senescence, oxidative stress, ferroptosis and pyroptosis, and modulate macrophage polarisation toward an anti-inflammatory phenotype [5,19]. These effects contribute to cartilage preservation and tissue repair in OA, RA and IVDD. In addition, advances in producer cell priming, next-generation producer cell engineering, precision EV engineering, biomaterial-assisted delivery and personalised MSC-EV therapy are accelerating the clinical translation of MSC-EVs. In this review, we summarise the current understanding of the source-specific properties, molecular mechanisms, therapeutic applications, engineering strategies, and clinical progress of MSC-EVs in cartilage regeneration and musculoskeletal disorders while highlighting the key challenges and future directions for their successful clinical translation.

## 2. MSC-EVs in Cartilage Regeneration: Mechanisms and Therapeutic Promise

Exosomes are nanosized extracellular vesicles (EVs) that function as natural intercellular delivery systems enriched with a wide spectrum of bioactive molecules, including microRNAs (miRNAs), long non-coding RNAs (lncRNAs), proteins, lipids and signalling mediators [1,2]. Among these cargos, miRNAs are especially important because they regulate gene expression post-transcriptionally by binding to complementary sequences in target mRNAs, leading to either translational repression or mRNA degradation.

**Figure 1:**
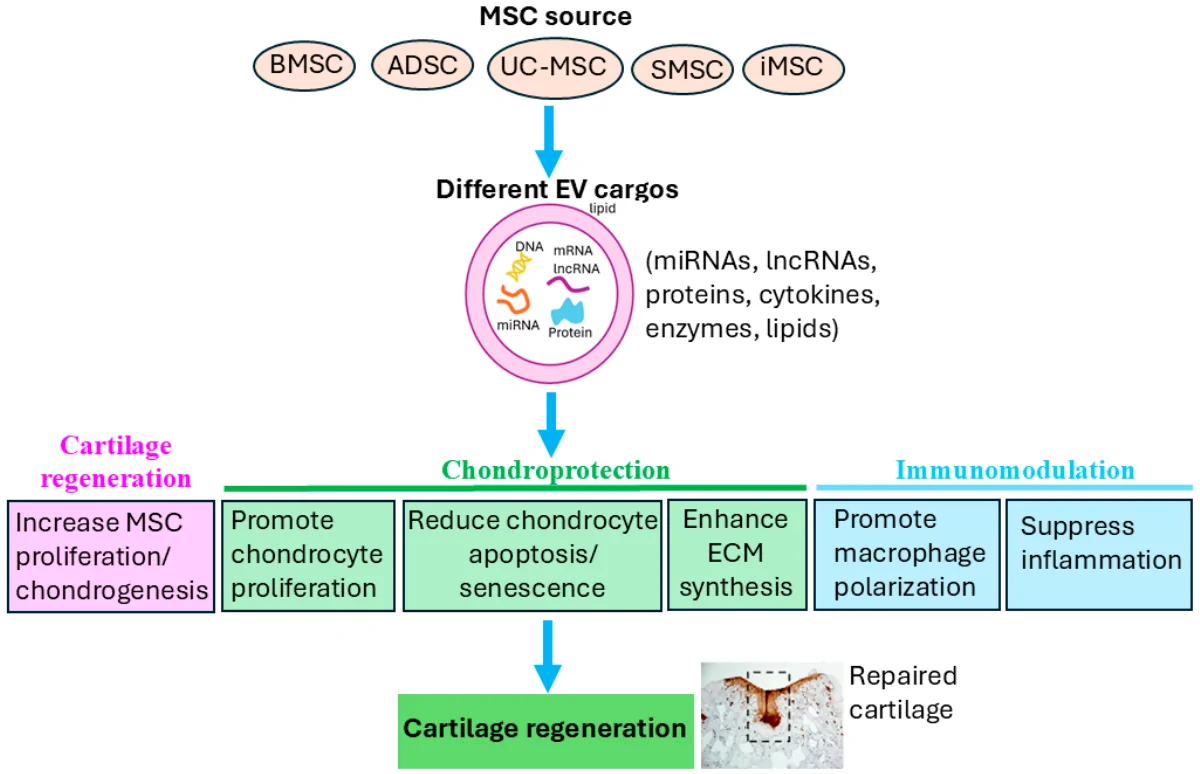
**Therapeutic mechanisms of extracellular vesicles (EVs) derived from different mesenchymal stromal/stem cell (MSC) sources in cartilage regeneration.** EVs can be isolated from multiple MSC sources, including bone marrow-derived MSCs (BMSCs), adipose-derived MSCs (ADSCs), umbilical cord-derived MSCs (UC-MSCs), synovium-derived MSCs (SMSCs) and induced pluripotent stem cell-derived MSCs (iMSCs). Although EVs from these sources contain distinct molecular cargos, including microRNAs (miRNAs), long non-coding RNAs (lncRNAs), proteins, cytokines and lipids, they promote cartilage repair and regeneration through multiple mechanisms. These include enhancing MSC proliferation and chondrogenic differentiation, promoting chondrocyte proliferation, reducing chondrocyte apoptosis and senescence, enhancing extracellular matrix (ECM) synthesis, promoting macrophage polarisation toward the anti-inflammatory M2 phenotype and suppressing inflammatory responses.

At the molecular level, exosomal miRNAs directly modulate key signalling pathways involved in cartilage degeneration and regeneration. Exosomal miR-125a-5p derived from bone marrow-derived MSCs (BMSC) promotes chondrocyte migration and attenuates cartilage degeneration by targeting *E2F2*. This is accompanied by increased expression of chondrogenic markers such as collagen type II (COL2A1), aggrecan and SOX9, along with reduced levels of the catabolic enzyme MMP-13, collectively promoting anabolic cartilage remodelling and maintaining the chondrocyte phenotype [20]. Similarly, exosomes derived from human umbilical cord MSCs (UC-MSCs) alleviate oxidative stress and inhibit chondrocyte apoptosis through regulation of the miR-100-5p/NOX4 signalling axis. By suppressing NOX4-mediated reactive oxygen species (ROS) production, these exosomes preserve chondrocyte viability and maintain cartilage integrity under inflammatory and degenerative conditions [21] (Figure 1 and Figure S1). These findings highlight that exosomal miRNAs play a key role in cartilage repair by simultaneously suppressing catabolic pathways and activating regenerative signalling networks.

Beyond miRNAs, MSC-derived exosomes also transport abundant lncRNAs that contribute substantially to cartilage regeneration and joint homeostasis. Exosomal lncRNAs regulate diverse biological processes involved in cartilage repair, including cell proliferation, apoptosis, ECM synthesis, inflammation and osteochondral regeneration (Figure 1). Exosomal lncRNA H19 derived from UC-MSCs significantly enhances osteochondral regeneration through modulation of the miR-29b-3p/FoxO3 signalling axis. Exosomes overexpressing lncRNA H19 enhance durable cartilage repair upon intra-articular injection. Mechanistically, H19 promotes chondrocyte migration and ECM secretion while simultaneously suppressing apoptosis and cellular senescence, thereby improving cartilage repair quality [22].

In addition to directly regulating chondrocytes, MSC-EVs enhance the regenerative potential of endogenous stem and progenitor cells within the joint microenvironment. Human umbilical cord Wharton’s jelly MSC-EVs (WJ-MSC-EVs) improve cartilage repair following microfracture treatment by enhancing chondrocyte viability and stimulating BMSC proliferation and chondrogenesis via the ITGB1/TGF-β/Smad2/3 signalling pathway [23]. Likewise, umbilical cord-derived MSC-EVs (UC-MSC-EVs) enhance BMSC proliferation, migration and chondrogenesis by delivering miR-181c-5p, which suppresses SMAD7 and thereby strengthens BMP2 signalling [24] (Figure S1). These studies highlight the ability of MSC-EVs to activate the body’s own regenerative pathways and recruit repair cells, ultimately enhancing the structural and functional recovery of cartilage tissue.

Another key therapeutic advantage of MSC-EVs is their strong immunomodulatory and anti-inflammatory effects (Figure 1). Since chronic inflammation is a major driver of cartilage breakdown, MSC-EVs can help regulate inflammation within the joint microenvironment. Hoffa’s fat pad MSC-EVs suppress the expression of catabolic enzymes such as MMP3 and MMP13 in chondrocytes while simultaneously reducing inflammatory mediators including CCL5 and COX2 in synovial fibroblasts. In parallel, these EVs enhance IL-10 secretion and promote M2-type anti-inflammatory macrophage polarisation [25]. Similarly, MSC-derived exosomes increase infiltration of CD163^+^ M2 macrophages while reducing CD86^+^ M1 macrophages and pro-inflammatory cytokines including IL-1β and TNF-α. Through these coordinated immunoregulatory effects, MSC-EVs create a pro-regenerative microenvironment that supports cartilage preservation and tissue remodelling [26] (Figure S1).

The therapeutic efficacy of MSC-EVs has also been validated in clinically relevant large animal models. In these models, MSC-EVs significantly enhance the repair of cartilage and subchondral bone, leading to improved tissue structure, better histological outcomes and stronger biomechanical properties [27]. Notably, exosome-treated defects often exhibit near-complete restoration of hyaline cartilage with seamless integration into surrounding native tissue and appropriate ECM organisation, whereas untreated controls typically develop fibrocartilaginous repair tissue with weaker mechanical properties [28]. These findings strongly support the potential of MSC-EVs for clinical translation in cartilage regeneration.

Comparable to MSC transplantation, MSC-EVs achieve therapeutic outcomes while avoiding many limitations associated with live-cell therapies [29]. Both adipose-derived and umbilical cord-derived MSC-EVs have shown strong cartilage repair effects, although the extent of regeneration can vary depending on the EV source, disease model, delivery method and experimental conditions. However, some studies suggest that EV treatment leads to the formation of fibrocartilage rather than true hyaline cartilage [30]. These differences are likely due to variations in EV isolation methods, cargo composition, dosing strategies, scaffold use and injury models.

In cartilage regeneration, MSC-EVs orchestrate multiple reparative processes by enhancing stem cell recruitment and differentiation, promoting chondrocyte proliferation and migration, stimulating ECM synthesis, suppressing inflammatory responses and inhibiting apoptosis and oxidative stress. Importantly, MSC-EVs also facilitate the regeneration of hyaline cartilage rather than fibrocartilage, thereby improving the structural and functional quality of repaired tissue. Through these coordinated effects, MSC-EVs help restore cartilage homeostasis and improve osteochondral repair, highlighting their strong potential as next-generation cell-free therapy for cartilage repair and osteochondral regeneration (Figure 1).

## 3. MSC-EVs in Osteoarthritis: Mechanisms and Therapeutic Effects

Osteoarthritis (OA) is a progressive degenerative joint disease characterised by cartilage breakdown, chronic inflammation, pain and subchondral bone remodelling, ultimately leading to substantial functional decline. Despite its growing clinical burden, effective disease-modifying therapies that halt cartilage degeneration and restore damaged tissue remain lacking. MSC-EVs have emerged as promising cell-free therapeutics for OA owing to their ability to modulate the joint microenvironment and protect cartilage. Through the transfer of bioactive cargos, including miRNAs, lncRNAs, proteins, lipids and cytokines, MSC-EVs regulate key pathological and regenerative processes, including inflammation, oxidative stress, chondrocyte apoptosis, extracellular matrix degradation, angiogenesis, subchondral bone remodelling and tissue regeneration, thereby offering a multifaceted approach to mitigating OA progression and promoting cartilage repair [31].

### 3.1. Promotion of Chondrocyte Proliferation, Migration and Phenotype Maintenance

**Figure 2:**
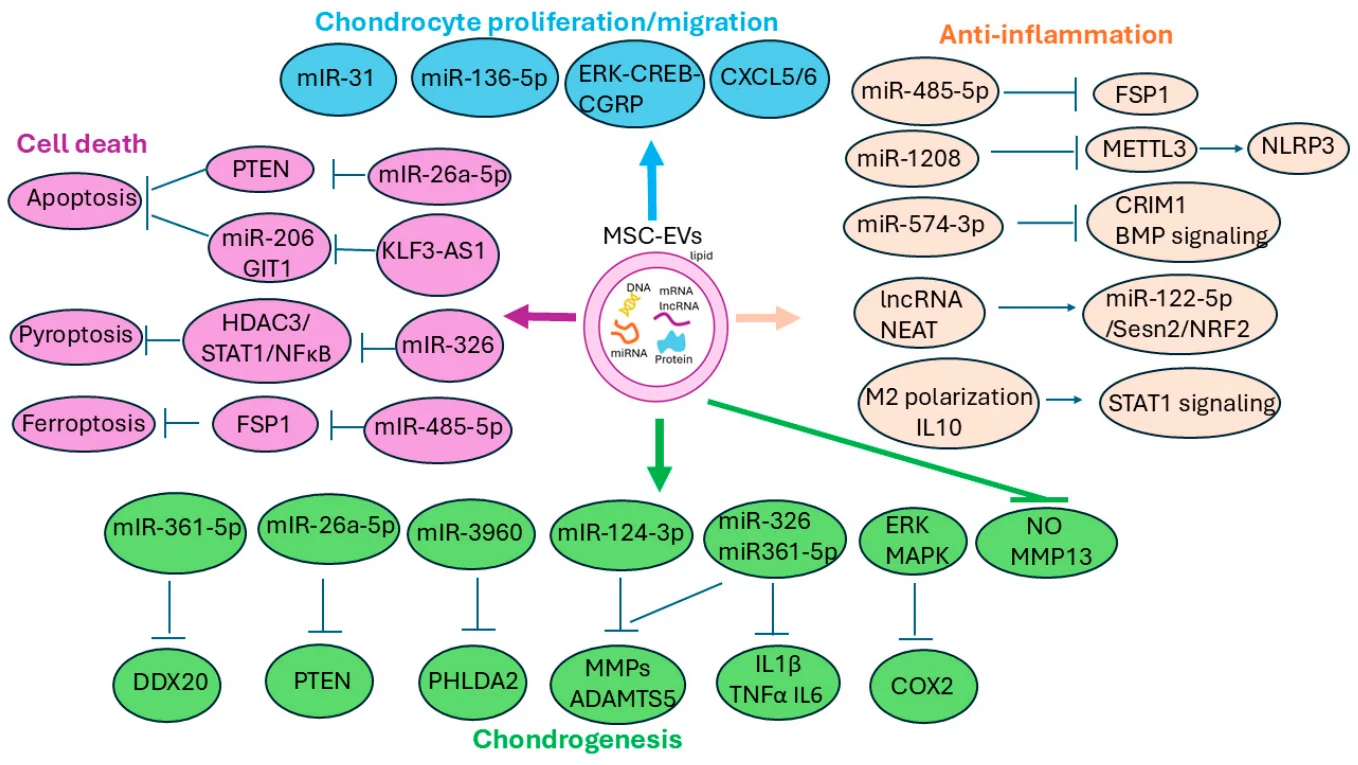
**MSC-EVs modulate cell fate, chondrogenesis and inflammation to inhibit the progression of osteoarthritis.** In osteoarthritis (OA), MSC-derived extracellular vesicles (EVs) promote chondrocyte proliferation, migration, chondrogenesis and extracellular matrix (ECM) synthesis while inhibiting apoptosis, pyroptosis, ferroptosis, inflammation and matrix degradation. These therapeutic effects are mediated by EV-derived miRNAs and lncRNAs that regulate multiple signalling pathways, thereby preserving cartilage homeostasis, promoting tissue repair and facilitating cartilage regeneration.

MSC-EVs actively support cartilage repair by promoting chondrocyte proliferation and migration to maintain their functional phenotype. These coordinated cellular responses promote ECM synthesis and enhance cell recruitment to injury sites to restore normal cartilage structure. At the molecular level, MSC-EVs stimulate chondrocyte expansion and motility through specific miRNA-mediated signalling pathways. Synovial MSC-derived EVs deliver miR-31, which activates the KDM2A/E2F1/PTTG1 axis to drive chondrocyte proliferation and migration [32]. Similarly, BMSC-derived exosomal miR-136-5p enhances migratory capacity and protects against cartilage degeneration [33]. In addition, dental pulp stem cell (DPSC)-derived EVs promote chondrocyte proliferation and migration while simultaneously reducing inflammation via the ERK-CREB-CGRP signalling pathway [34]. Complementing these effects, EVs from Wharton’s jelly MSCs (WJMSC-EVs) enhance proliferation and chondrogenesis through miRNA-mediated activation of calcium signalling, NOTCH and ECM–receptor interaction pathways [35]. In a meniscus defect model, synovial MSC-EV injection enhances meniscus regeneration and restores tissue repair. MSC-EVs enhance chondrocyte and synovial MSC growth and migration via upregulation of CXCL5 and CXCL6. Blocking CXCL5/CXCL6 or antagonising their receptor CXCR2 abolishes these effects, confirming the essential role of this signalling axis [36] (Table 1, Figure 2). ADSC-EVs mitigate cartilage degeneration in osteoarthritic rodents by suppressing multiple inflammatory cytokines and upregulating FGF-18 [37]. BMSC-EVs enhance chondrocyte proliferation, reduce apoptosis and suppress inflammatory cytokines including TNF-α, IL-6 and IL-1β by inhibiting NF-κB p65 phosphorylation in vitro. In vivo, they attenuate cartilage erosion, synovial hyperplasia and inflammatory infiltration, demonstrating overall protective effects in OA progression [38] (Table 1).

### 3.2. Regulation of Chondrocyte Cell Death

MSC-EVs play a key role in maintaining chondrocyte viability by targeting multiple interconnected forms of regulated cell death, including apoptosis, pyroptosis and ferroptosis, as well as key cellular stress pathways that contribute to OA progression. Under inflammatory conditions, MSC-EVs first act to suppress apoptosis and pyroptosis, two major contributors to chondrocyte loss. SMSC-EVs suppress apoptosis, thereby ameliorating cartilage damage in OA by delivering miR-26a-5p into chondrocytes to inhibit PTEN [39]. MSC-derived exosomal KLF3-AS1 suppresses IL-1β-induced chondrocyte apoptosis while facilitating cartilage repair by regulating the KLF3-AS1/miR-206/GIT1 axis in a rat model of OA [40]. BMSC-derived exosomes deliver miR-326, which inhibits pyroptosis by targeting HDAC3 and downregulating the STAT1/NF-κB signalling pathway [41]. In addition, MSC-EVs also regulate other forms of programmed cell death. Notably, BMSC-derived exosomes containing lncRNA SNHG7 suppress ferroptosis, inflammation and cellular damage by modulating the miR-485-5p/FSP1 axis [42] (Table 1, Figure 2).

At the organelle level, mitochondrial dysfunction is a key driver of chondrocyte degeneration. ADSC-EVs counteract this by activating the PINK1/Parkin pathway to promote mitochondrial autophagy and restore mitochondrial homeostasis, which in turn reduces inflammation and cell death [43]. MSC-EVs mitigate upstream cellular stressors that trigger these death pathways. UC-MSC-derived EVs effectively reduce oxidative stress and cellular senescence while enhancing mitochondrial function, thereby creating a protective intracellular environment that supports chondrocyte survival [44,45] (Table 1, Figure 2).

### 3.3. ECM Preservation and Cartilage Regeneration

A key therapeutic role of MSC-EVs is maintaining extracellular matrix (ECM) integrity, which is essential for cartilage homeostasis and normal biomechanical function. In osteoarthritic and inflammatory microenvironments, pro-inflammatory cytokines such as IL-1β stimulate ECM degradation by upregulating catabolic enzymes, including matrix metalloproteinases (MMPs) and members of the ADAMTS family, leading to the breakdown of collagen II and aggrecan, the major structural components of cartilage ECM. MSC-EVs counteract these degenerative processes by restoring the expression of key ECM molecules, particularly collagen II and aggrecan, thereby promoting matrix synthesis and cartilage repair [46,79] (Figure 2). MSC-EVs suppress the expression and activity of catabolic mediators associated with cartilage degeneration. Embryonic stem cell-derived MSC (ESC-MSC)-derived EVs not only preserve collagen II synthesis but also markedly inhibit ADAMTS5 expression, thereby reducing aggrecan degradation and maintaining ECM stability [47]. Exosomal miR-361-5p alleviates chondrocyte injury through targeting DDX20 and suppressing NF-κB activation [48] (Table 1). MSC-EVs downregulate MMP-13 and other matrix-degrading enzymes while enhancing anabolic chondrogenic markers such as SOX9, further supporting cartilage matrix regeneration and chondrocyte phenotype maintenance. Through these combined anabolic and anti-catabolic effects, MSC-EVs help re-establish ECM homeostasis, attenuate cartilage degeneration and enhance the regenerative capacity of damaged cartilage tissue. Mechanistically, these protective effects are often mediated by EV-delivered microRNAs. One clear example is EV-miR-3960, which suppresses the SDC1/Wnt/β-catenin pathway, a signalling axis known to promote chondrocyte catabolism and matrix breakdown. By inhibiting this pathway, miR-3960 helps preserve cartilage structure [49]. miR-124-3p exerts chondroprotective effects by downregulating catabolic enzymes such as MMP13 and ADAMTS5 while promoting ECM synthesis by upregulating COL2A1 [50] (Table 1, Figure 2). Dental pulp stem cell (DPSC)-derived EVs promote cartilage regeneration and relieve pain in temporomandibular joint osteoarthritis (TMJOA) [34].

However, not all EV-associated signals are purely protective; some require fine balance. Exosomes carrying Wnt5a/b activate YAP signalling, which paradoxically promotes chondrocyte proliferation but reduces ECM secretion. This trade-off could lead to matrix-poor, hypercellular tissue if unopposed. Notably, exosomal miR-140-5p helps restore extracellular matrix (ECM) homeostasis, likely by targeting pro-inflammatory and catabolic factors. This effect is particularly evident in synovium-derived MSC-EVs, which are enriched in miR-140-5p. These exosomes promote chondrocyte proliferation without impairing extracellular matrix (ECM) deposition, overcoming a limitation observed with Wnt5a/b/YAP activation alone. As a result, SMSC-derived exosomes show strong therapeutic potential for cartilage repair by supporting both cell expansion and proper matrix formation [51] (Table 1). These findings show that MSC-EVs coordinate cartilage repair through a well-balanced network of signalling pathways. This coordinated regulation keeps chondrocyte proliferation, differentiation and ECM maintenance in balance, supporting effective cartilage regeneration while preserving matrix integrity and tissue homeostasis.

### 3.4. Immunomodulatory and Anti-Inflammatory Effects

Beyond their direct effects on matrix protection, MSC-EVs also exert strong immunomodulatory functions that are central to their therapeutic benefits in OA. Chronic low-grade inflammation in OA is driven by pro-inflammatory cytokines that further accelerate ECM degradation. MSC-EVs counter this inflammatory environment by reducing levels of IL-1β, IL-6 and TNF-α while increasing the anti-inflammatory cytokine IL-10. This cytokine shift not only dampens catabolic signalling but also reshapes the immune landscape within the joint. Similarly, UC-MSC-EVs prevent OA progression by suppressing the secretion of pro-inflammatory factors while enhancing the expression of cartilage-specific ECM markers, including COL2A1 and aggrecan [52] (Table 1, Figure 2).

EV-delivered miR-1208 directly targets METTL3 to reduce m6A methylation of NLRP3 mRNA, which attenuates NLRP3-mediated inflammatory signalling and promotes cartilage homeostasis [80] (Figure 2). MSC-derived exosomal KLF3-AS1 suppresses IL-1β-induced inflammatory responses and facilitates cartilage repair through the KLF3-AS1/miR-206/GIT1 axis in a rat model of OA [40]. Synovial MSC-EVs also attenuate chondrocyte inflammation via miR-129-5p targeting HMGB1 [53] (Table 1, Figure 2).

Beyond miRNAs, lncRNAs and circRNAs within MSC-EVs further expand this regulatory landscape by functioning as competing endogenous RNAs (ceRNAs) that modulate miRNA availability and downstream signalling pathways. Exosomal lncRNA SNHG7 suppresses inflammation by modulating the miR-485-5p/FSP1 axis [42], while lncRNA NEAT1 activates the miR-122-5p/Sesn2/NRF2 signalling axis to enhance autophagy and cellular stress resistance [54]. Likewise, circYAP1 alleviates OA progression by regulating the miR-21/TLR7 pathway, thereby suppressing inflammation [55]. Additionally, circHIPK3 acts as a molecular sponge for miR-124-3p, leading to upregulation of MYH9 and contributing to cytoskeletal stability and cellular function [56] (Table 1, Figure 2). These findings point to a coordinated RNA-mediated regulatory network through which MSC-EVs suppress inflammation, regulate chondrocyte function and promote cartilage repair, highlighting their therapeutic potential in osteoarthritis.

A key mechanism underlying this shift is the reprogramming of macrophages. Macrophages in the OA synovium often display a pro-inflammatory M1 phenotype, contributing to synovitis and cartilage breakdown. EVs derived from UC-MSCs promote a switch toward the anti-inflammatory M2 phenotype, an effect mediated in part by modulation of STAT1 signalling [57,58]. M2-polarised macrophages subsequently secrete IL-10 and other reparative factors, reinforcing ECM protection and resolving inflammation [58,81]. Furthermore, TGF-β1-stimulated BMSC-EVs ameliorate cartilage damage by repressing M1 polarisation while promoting M2 polarisation of synovial macrophages via miR-135b targeting MAPK6 [59] (Table 1, Figure 2).

At the molecular level, MSC-EVs suppress central inflammatory signalling hubs, notably the NF-κB [48] and MAPK pathways [61]. Inhibition of these pathways decreases pro-inflammatory cytokines such as TNF-α and IL-6, thereby reducing the IL-1β-induced inflammatory response. By suppressing NF-κB and MAPK, MSC-EVs act as a dual brake, attenuating inflammation while directly limiting ECM destruction. This aligns with earlier observations that EVs restore collagen II and aggrecan in IL-1β-treated cartilage [46,79]. At the tissue level, this results in reduced synovial inflammation, less macrophage infiltration, and decreased osteophyte formation, all of which are key features of OA progression [52]. Thus, MSC-EV-mediated immunomodulation contributes to structural joint protection rather than merely providing symptomatic relief. ADSC-EVs inhibit synovial inflammation in osteoarthritic rodent models, an effect associated with reduced inflammatory cytokine levels and increased FGF18 expression [37] (Table 1).

Interestingly, not all EV populations show the same level of efficacy. Comparative studies suggest that, compared with matrix-bound vesicles such as microvesicles retained within connective tissue, exosomes exhibit stronger anti-inflammatory and cartilage-protective effects. This may reflect differences in surface receptor display, miRNA cargo selectivity or stability in synovial fluid [82].

These findings indicate that MSC-EVs orchestrate a coordinated immunomodulatory response by regulating the cytokine environment, reprogramming macrophages and suppressing NF-κB/MAPK signalling pathways, ultimately reducing synovial inflammation.

### 3.5. Key Signalling Pathways Mediated by MSC-EVs

MSC-EVs regulate multiple signalling pathways involved in OA progression in a coordinated manner that suppresses catabolic activity, promotes anabolic processes and suppresses inflammation. The NF-κB pathway, a master driver of inflammation and matrix degradation, is directly inhibited by EV-delivered microRNAs including miR-326 and miR-361-5p [41,48] (Table 1). This suppression reduces the transcription of pro-inflammatory cytokines including IL-1β, TNF-α and IL-6, and matrix-degrading enzymes including MMPs and ADAMTS5, linking back to the immunomodulatory and ECM-protective functions.

Similarly, the Wnt/β-catenin pathway, which promotes chondrocyte catabolism, hypertrophy and osteophyte formation when aberrantly activated, is suppressed by MSC-EVs to prevent cartilage degradation [49,83]. Notably, EV-miR-3960 protects against chondrocyte apoptosis and ECM degradation and mitigates chondrocyte injury in osteoarthritis, by downregulating PHLDA2 via the SDC1/Wnt/β-catenin signalling axis [49] (Table 1, Figure 2), reinforcing the theme that miRNA cargo is a key effector. Wnt/β-catenin inhibition also complements NF-κB suppression, as both pathways converge on genes involved in inflammation and matrix breakdown.

In OA, the PI3K-Akt pathway plays a dual role, supporting chondrocyte survival and ECM synthesis while also contributing to pathological hypertrophy under certain conditions. MSC-EVs modulate this pathway through specific miRNAs, including miR-let-7a-5p, miR-100-5p, miR-122-5p, miR-148a-3p and miR-486-5p, shifting the balance toward protective outcomes. This effect is enhanced by synergy with M2 macrophage polarisation [58].

OA is closely linked to dysregulation of the BMP signalling pathway, which plays essential roles in cartilage development, chondrocyte differentiation, ECM maintenance and tissue repair. Exosomal miR-574-3p derived from ADSCs regulates the CRIM1/BMP signalling axis to suppress chondrocyte hypertrophy and inflammatory response [60] (Table 1, Figure 2).

The MAPK pathway, including p38, JNK and ERK, is another inflammatory hub modulated by EVs to reduce inflammation and cartilage degeneration while enhancing cartilage repair. MAPK inhibition often co-occurs with NF-κB suppression, as both pathways are activated by IL-1β and TNF-α, and both are targeted by common EV cargoes. Amniotic fluid-derived MSC-EVs (AF-MSC) exert anti-inflammatory effects against IL-1β and TNF-α by significantly suppressing ERK phosphorylation in the MAPK pathway, thereby reducing downstream production of pro-inflammatory COX2 products to restore the expression of anabolic genes [61]. BMSC-EVs attenuate IL-1β-induced chondrocyte injury by restoring cell viability, suppressing apoptosis and stabilising mitochondrial membrane potential through AKT/ERK/AMPK signalling [62]. MSC-EVs alleviate temporomandibular joint osteoarthritis (TMJOA) by suppressing IL-1β-induced nitric oxide and MMP13 production to attenuate inflammation and enhance s-GAG synthesis [84] (Figure 2).

MSC-EVs can modulate YAP signalling during cartilage repair and osteoarthritis treatment. As a key mechanotransduction and pro-proliferative pathway, YAP signalling plays an important role in cartilage regeneration and tissue homeostasis. MSC-EVs regulate Hippo-YAP pathway activity through multiple mechanisms, including the delivery of miRNAs, proteins and lipid mediators that fine-tune signalling in chondrocytes and surrounding joint tissue, thereby promoting cartilage repair while maintaining ECM integrity. Exosome-delivered Wnt5a and Wnt5b activate YAP signalling through the non-canonical Wnt pathway, thereby enhancing chondrocyte proliferation and migration. However, this activation was accompanied by a significant reduction in ECM secretion [51]. Therefore, the regenerative effects of YAP signalling depend on precise context-dependent modulation. In fact, excessive or unbalanced YAP activation may impair ECM synthesis and promote dedifferentiation or hypertrophic changes in chondrocytes. Therefore, beneficial YAP activity requires precise regulation. MSC-EVs achieve this balance through co-delivered regulatory miRNAs such as miR-140-5p, which modulate YAP-associated signalling via targets including RalA signalling [51]. This integrated regulation enables enhanced cellular proliferation and migration while preserving ECM synthesis and cartilage-specific phenotypes. Moreover, intra-articular administration of these exosomes effectively attenuate cartilage degeneration and prevent OA progression in a rat model, highlighting their therapeutic potential for cartilage regeneration and OA treatment. In addition, BMSC-EVs also promote cartilage regeneration in TMJOA through autotaxin–YAP signalling [63] (Table 1).

Therapeutically, exosomes can restore cartilage homeostasis through multiple complementary mechanisms. They promote chondrocyte survival and proliferation, stimulate synthesis of cartilage-specific ECM components such as collagen type II and aggrecan, inhibit catabolic enzymes including MMP13 and ADAMTS5, and suppress inflammatory mediators such as IL-1β, TNF-α and COX2. In addition, exosomes regulate macrophage polarisation toward anti-inflammatory M2 phenotypes, thereby creating a regenerative joint microenvironment conducive to cartilage repair (Table 1, Figure 2).

### 3.6. Effects on Subchondral Bone and Whole Joint Environment

Beyond their direct effects on chondrocytes, MSC-EVs also exert multi-tissue protective functions across the entire joint microenvironment, particularly in subchondral bone remodelling and osteochondral crosstalk. At the level of subchondral bone, MSC-EVs regulate bone homeostasis by modulating osteoclast activity. EV-delivered let-7a-5p suppresses osteoclastogenesis, thereby preserving subchondral bone integrity and preventing excessive bone resorption [85]. In parallel, MSC-EVs contribute to the restoration of balanced bone remodelling by promoting osteoblast activity while inhibiting pathological angiogenesis, a key feature associated with OA progression and pain sensitization [86]. Importantly, these molecular and structural improvements translate into functional benefits at the whole-joint level. In both TMJOA [84] and knee OA models [87], MSC-EVs attenuate inflammation and pain while improving overall joint function. This highlights their capacity to simultaneously target cartilage, bone and synovial components. MSC-EVs act as integrative regulators of the joint microenvironment, coordinating anti-resorptive, anti-angiogenic and anti-inflammatory effects to promote joint homeostasis and functional recovery.

These findings suggest that MSC-EVs regulate OA progression and cartilage repair through coordinated modulation of multiple signalling pathways. They suppress catabolic and inflammatory pathways, including NF-κB, Wnt/β-catenin and MAPK signalling, while also modulating mechanosensitive and proliferative pathways such as YAP signalling in a context-dependent manner. At the same time, anabolic and pro-survival pathways, including PI3K-Akt, ERK and AMPK signalling, are finely regulated to maintain cellular homeostasis, support chondrocyte survival and promote ECM synthesis. Through the delivery of bioactive miRNAs, lncRNAs and proteins, MSC-EVs regulate chondrocyte function, immune responses and subchondral bone remodelling. Together, these integrated effects contribute to reduced inflammation, preservation of ECM integrity, inhibition of cartilage degeneration and enhanced tissue regeneration, highlighting MSC-EVs as a promising multifaceted therapeutic strategy for osteoarthritis treatment.

## 4. Immunomodulatory and Tissue-Protective Effects of MSC-EVs in Rheumatoid Arthritis

Rheumatoid arthritis (RA) is a chronic autoimmune disease characterised by persistent synovial inflammation, cartilage degradation and progressive bone erosion. MSC-EVs show therapeutic potential as cell-free agents by delivering bioactive cargos that exert immunomodulatory, anti-inflammatory and tissue-protective effects.

**Figure 3:**
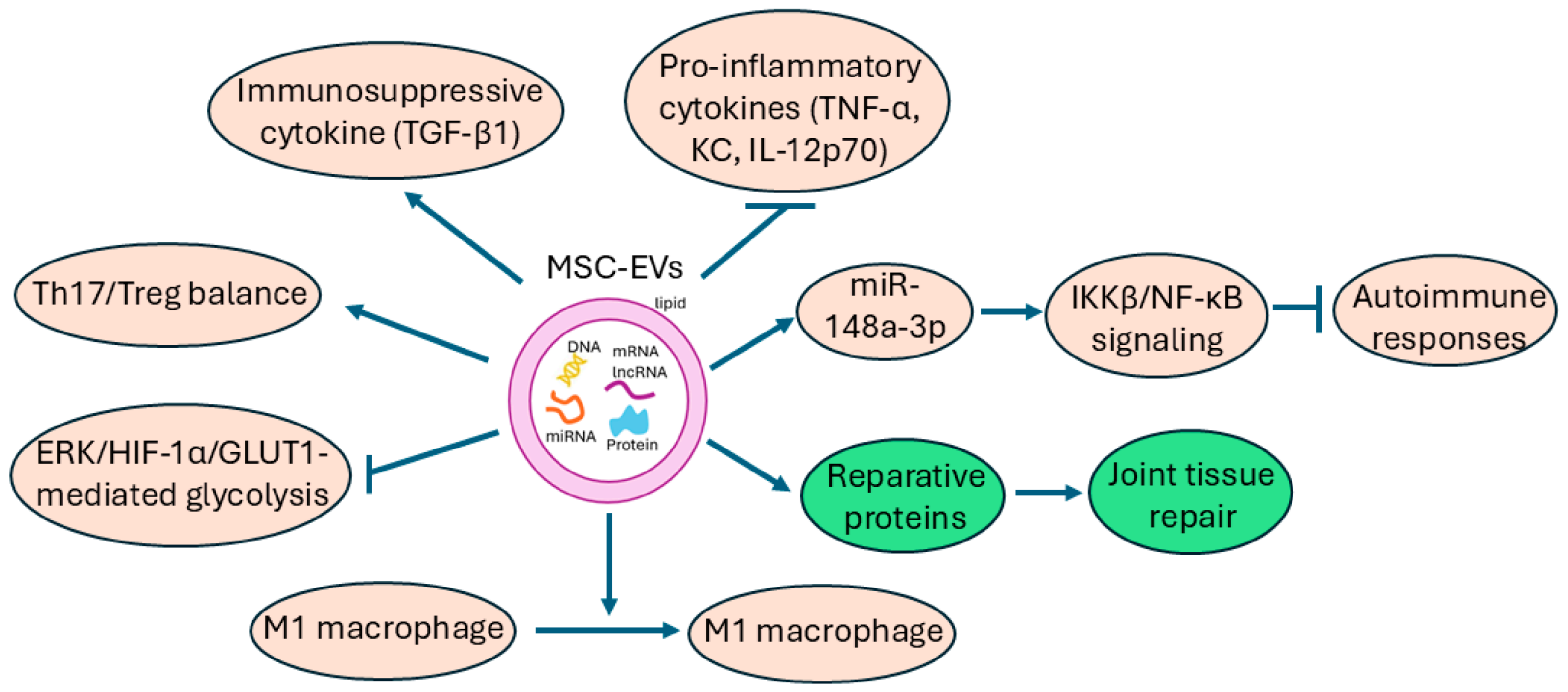
**Therapeutic mechanisms of MSC-derived extracellular vesicles (MSC-EVs) in rheumatoid arthritis (RA).** MSC-EVs attenuate RA by promoting M1-to-M2 macrophage polarisation, suppressing inflammatory glycolysis, restoring Th17/Treg balance, enhancing immunosuppressive cytokine production (TGF-β1), reducing pro-inflammatory cytokines and promoting joint tissue repair and regeneration.

A central mechanism underlying the therapeutic efficacy of MSC-EVs in RA is their ability to regulate macrophage polarisation. MSC-EVs promote the transition of pro-inflammatory M1 macrophages toward the anti-inflammatory M2 phenotype, thereby reducing synovial inflammation and attenuating disease progression [64]. This immunoregulatory effect can be further enhanced through engineered EV approaches. ADSC-derived EVs loaded with icariin not only promote M2 macrophage polarisation but also suppress ERK/HIF-1α/GLUT1-mediated glycolysis, a key metabolic pathway that supports inflammatory macrophage activation. This dual modulation of immune phenotype and cellular metabolism effectively reduces inflammation and preserves cartilage integrity in collagen-induced RA rat models [65] (Table 1, Figure 3).

In addition to macrophage regulation, MSC-EVs also regulate adaptive immune responses, particularly T cell homeostasis. Gingiva-derived MSC-EVs (GMSC-EVs) preferentially accumulate in inflamed joints and restore the balance between pro-inflammatory Th17 cells and regulatory T cells (Treg). Mechanistically, this effect is largely mediated by miR-148a-3p through modulation of the IKKβ/NF-κB signalling pathway, ultimately suppressing autoimmune responses and slowing RA progression [11]. Furthermore, exosomes derived from immortalised MSCs primed with RA serum enhance TGF-β1 production and promote Th2 and M2 polarisation while simultaneously suppressing pro-inflammatory cytokines, including TNF-α, K and IL-12p70, further reinforcing their broad immunosuppressive properties [66] (Table 1, Figure 3).

Beyond immune modulation, MSC-EVs also contribute directly to joint tissue repair and regeneration. BMSC-derived exosomes alleviate RA symptoms in CIA models, likely through the delivery of proteins associated with cell adhesion, tissue remodelling and regenerative processes, thereby supporting structural recovery of damaged joints [67] (Table 1, Figure 3). These findings suggest that MSC-EVs exert therapeutic effects in RA through the coordinated modulation of innate and adaptive immune responses, metabolic reprogramming and tissue repair processes. Owing to their multifaceted mechanisms of action, MSC-EVs represent promising cell-free therapeutic candidates for the treatment of inflammatory RA.

## 5. MSC-EVs in Intervertebral Disc Degeneration: Mechanisms and Therapeutic Advances

Intervertebral disc degeneration (IVDD) is a major cause of low back pain, characterised by progressive nucleus pulposus degeneration, annulus fibrosus disruption, extracellular matrix (ECM) degradation and chronic inflammation. MSC-EVs have emerged as promising cell-free therapeutics for IVDD by delivering bioactive cargos, including miRNAs, proteins and lipids, to modulate the disc microenvironment. Through suppressing inflammation and apoptosis while promoting cell proliferation and ECM synthesis, MSC-EVs may enhance disc regeneration and restore spinal function.

### 5.1. Modulation of Cell Death, Senescence, and Mitochondrial Homeostasis

MSC-EVs play a crucial role in preserving nucleus pulposus cell (NPC) viability and delaying IVDD by regulating cell death, senescence and mitochondrial homeostasis. In IVDD, where inflammatory stress, oxidative damage and mechanical compression accelerate disc cell loss, MSC-EVs provide multifaceted protection that helps maintain cellular function and ECM integrity.

**Figure 4:**
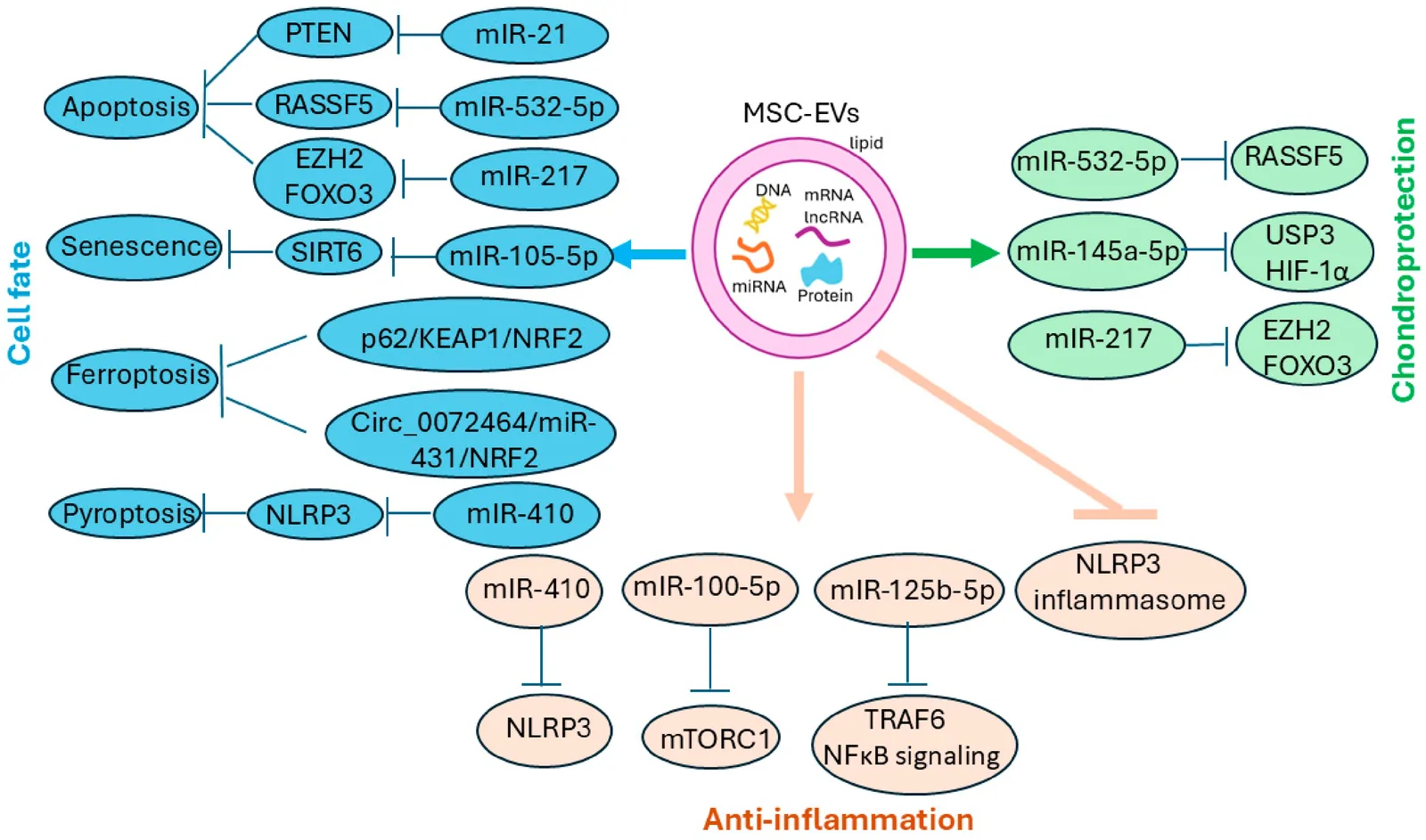
**MSC-EVs suppress cell death and inflammation and promote chondroprotection in intervertebral disc degeneration (IVDD).** In intervertebral disc degeneration (IVDD), MSC-derived EVs inhibit apoptosis, senescence, ferroptosis and pyroptosis while preserving nucleus pulposus cell function. They also suppress inflammation. These therapeutic effects are mediated through EV-derived microRNAs (miRNAs), circular RNAs (circRNAs), and long non-coding RNAs (lncRNAs), which regulate multiple signalling pathways to improve NPC survival, restore ECM integrity and slow IVDD progression.

Ferroptosis, an iron-dependent form of regulated cell death characterised by excessive lipid peroxidation and oxidative stress, has recently been recognised as a critical pathological mechanism contributing to IVDD. Increasing evidence indicates that MSC-EVs can effectively counteract ferroptosis through the activation of antioxidant and cytoprotective signalling pathways, particularly the p62/KEAP1/NRF2 axis [68]. Activation of NRF2 promotes the transcription of multiple antioxidant and detoxification enzymes, thereby reducing reactive oxygen species (ROS) accumulation, limiting lipid peroxidation and protecting NPCs from oxidative damage. In addition to direct NRF2 activation, MSC-EVs also regulate ferroptosis through non-coding RNA-mediated mechanisms. The circ_0072464/miR-431/NRF2 regulatory axis enhances NRF2 expression by relieving miR-431-mediated suppression, resulting in reduced ferroptotic cell death, enhanced NPC proliferation and improved ECM synthesis [69] (Table 1, Figure 4). These effects contribute to the preservation of disc structure and maintenance of disc cellular homeostasis under degenerative conditions.

Beyond ferroptosis inhibition, MSC-EVs also exert anti-pyroptotic effects in IVDD. Pyroptosis is a pro-inflammatory form of programmed cell death closely associated with activation of the NLRP3 inflammasome. MSC-EVs suppress NLRP3 signalling through delivery of miR-410 to reduce pyroptosis-mediated NPC injury, inflammasome activation and inflammatory cytokine release [70] (Table 1, Figure 3). Through simultaneous suppression of oxidative stress, ferroptosis and inflammasome-driven pyroptosis, MSC-EVs establish a broad cytoprotective environment that supports NPC survival and disc regeneration. This highlights the central role of EV-mediated cell death modulation and redox regulation in maintaining intervertebral disc homeostasis. By coordinately regulating multiple forms of regulated cell death, antioxidant defences, mitochondrial function and inflammatory signalling, MSC-EVs represent a promising cell-free therapeutic strategy for preventing IVDD progression and promoting intervertebral disc tissue regeneration.

Human MSC-EVs have been shown to enhance cell viability in degenerated disc cells, suggesting their capacity to partially restore disc-like phenotypes and support tissue repair in degenerative IVDD environments [88]. EV-delivered microRNAs serve as key effectors of these protective actions. Exosomal miR-21 attenuates TNF-α-induced NPC apoptosis by targeting PTEN to activate the PI3K/Akt signalling pathway, a central pro-survival cascade that enhances cell resistance to inflammatory injury [71] (Table 1). Exosomal miR-532-5p inhibits apoptosis by targeting RASSF5 [72]. miR-217 suppresses apoptosis by targeting EZH2 to relieve transcriptional repression of FOXO3, a key regulator of cellular stress resistance and longevity [73] (Figure 4).

In addition, MSC-EVs also exert anti-senescence effects. iMSC-EVs carrying miR-105-5p activate the SIRT6 signalling pathway, which is associated with genomic stability, metabolic regulation and anti-ageing responses, thereby reversing nucleus pulposus cell senescence and improving disc cell function [12] (Figure 4). Furthermore, MSC-EVs alleviate oxidative stress-induced mitochondrial dysfunction by preserving mitochondrial membrane potential, reducing excessive reactive oxygen species (ROS) accumulation and limiting apoptosis under compressive stress conditions [74] (Table 1). These effects collectively help maintain mitochondrial integrity, a key determinant of NPC survival in the harsh hypoxic and mechanically loaded disc microenvironment. Therefore, MSC-EVs provide a coordinated protective network that integrates anti-apoptotic signalling, senescence reversal and mitochondrial stabilisation. Through these complementary mechanisms, they effectively preserve NPC function, slow disc degeneration and support regeneration of the intervertebral disc microenvironment.

### 5.2. ECM Homeostasis and Tissue Regeneration

ECM integrity is essential for maintaining intervertebral disc structure and function. During degeneration, excessive matrix degradation, fibrosis, and the loss of collagen II and aggrecan impair disc homeostasis, whereas MSC-EVs help restore ECM balance and promote regenerative remodelling. BMSC-derived exosomes regulate multiple signalling pathways involved in ECM preservation and tissue repair. Exosomal miR-532-5p inhibits ECM breakdown and fibrosis by targeting RASSF5 to attenuate degenerative signalling and preserve disc structural integrity [72]. BMSC-EVs suppress matrix degradation while enhancing regenerative responses in NPCs through the miR-145a-5p/USP31/HIF-1α signalling axis. Through regulation of HIF-1α-associated pathways, MSC-EVs maintain the hypoxic adaptation and metabolic homeostasis that are critical for NPC survival within the avascular disc microenvironment [75] (Table 1). Additional EV-delivered miRNAs further reinforce these protective effects. MSC-EVs attenuate ECM degradation via miR-217, which targets EZH2 to relieve its repression of FOXO3, thereby stimulating autophagy. This results in increased expression of collagen II and aggrecan, alongside reduced levels of MMP13 and ADAMTS5 [73] (Figure 4). Beyond individual miRNAs, MSC-EVs broadly regulate regenerative gene expression programmes in NPCs to promote cell proliferation, enhance synthesis of key ECM components such as collagen II and aggrecan, and restore ECM homeostasis [89].

Importantly, MSC-EVs not only stimulate anabolic matrix synthesis but also suppress catabolic pathways associated with IVDD progression. By reducing the expression of matrix-degrading enzymes and inflammatory mediators while enhancing chondrogenic and reparative signalling, MSC-EVs rebalance anabolic–catabolic activity within the disc microenvironment. These coordinated actions support maintenance of disc architecture, improve cellular viability and facilitate tissue regeneration. These findings demonstrate that MSC-EVs function as potent regulators of ECM preservation and regenerative remodelling in IVDD. Through integrated modulation of hypoxia adaptation, anti-fibrotic signalling, anabolic matrix synthesis and catabolic suppression, MSC-EVs provide a promising cell-free therapy for restoring intervertebral disc homeostasis and delaying degenerative progression [90].

### 5.3. Regulation of Inflammation and Immune Microenvironment

Inflammation drives IVDD by promoting NPC dysfunction, ECM degradation, oxidative stress and structural damage. MSC-EVs exert anti-inflammatory and immunomodulatory effects to restore disc homeostasis and attenuate degeneration. Induced MSC-derived EVs modulate macrophage–NPC interactions, promoting a more balanced immune microenvironment and reducing inflammatory signalling within degenerative discs. MSC-EVs reduce inflammasome activation and inflammatory cytokine release by suppressing NLRP3 signalling through delivery of miR-410 [70]. By regulating crosstalk between immune cells and disc cells, MSC-EVs limit chronic inflammatory activation that drives ECM breakdown and NPC apoptosis, partly through miR-100-5p-mediated suppression of mTORC1 signalling and regulation of glycolytic metabolic reprogramming [76]. These effects are particularly important because infiltrating and activated macrophages are increasingly recognised as major contributors to IVDD-associated inflammation and pain. MSC-EVs suppress activation of key inflammatory mediators and inhibit NLRP3 inflammasome signalling in NPCs. Inhibition of NLRP3 activation reduces inflammatory cytokine release, pyroptosis and downstream tissue injury, thereby slowing IVDD progression [77] (Table 1, Figure 3). Through suppression of inflammasome-mediated inflammatory cascades, MSC-EVs create a more protective microenvironment that supports NPC survival and matrix preservation.

The anti-inflammatory capacity of MSC-EVs is further reinforced through EV-mediated delivery of regulatory microRNAs. Exosomal miR-125b-5p modulates the TRAF6/NF-κB signalling pathway, which is a central regulator of inflammatory gene expression in degenerative disc disease. By suppressing NF-κB activation, MSC-EVs reduce the production of pro-inflammatory cytokines and catabolic mediators, thereby attenuating inflammation-induced ECM degradation and cellular injury [78] (Table 1, Figure 3).

MSC-EVs target multiple pathological processes in IVDD, including apoptosis, senescence, ferroptosis, pyroptosis, ECM degradation, and inflammation. Their multifaceted, cell-free therapeutic potential is supported by in vivo rat and rabbit models, in which intradiscal administration improves NPC survival, restores ECM integrity, and attenuates IVDD progression.

## 6. MSC-EV Clinical Applications in Cartilage Repair, OA, RA and IVDD

Despite strong preclinical evidence supporting the regenerative and immunomodulatory effects of MSC-EVs, their clinical translation for musculoskeletal disorders remains at an early stage. In cartilage repair and osteoarthritis (OA), preclinical studies consistently demonstrate that MSC-EVs promote cartilage matrix synthesis and regeneration while reducing inflammation, apoptosis, senescence and catabolic activity. They can also modulate the joint immune microenvironment, particularly by promoting macrophage polarisation toward reparative phenotypes. However, only a limited number of early-phase clinical trials are currently evaluating MSC-EV therapies for OA and cartilage repair, and robust clinical efficacy data remain unavailable (Table 2).

Translation to rheumatoid arthritis (RA) and intervertebral disc degeneration (IVDD) is even less advanced, with evidence largely restricted to in vitro and animal studies. In RA, MSC-EVs have shown potential to suppress synovial inflammation and regulate immune cell responses, whereas in IVDD they can reduce apoptosis, senescence, oxidative stress and extracellular matrix degradation while supporting tissue homeostasis. However, the therapeutic efficacy, optimal dosing and long-term safety of MSC-EVs in these indications remain to be established clinically.

Several barriers currently limit clinical translation, including: substantial variability in MSC sources, culture conditions and EV production; differences in isolation and purification methods; and the lack of standardised criteria for EV identity, purity, quality and potency. Limited scalability and challenges in establishing GMP-compliant manufacturing further complicate clinical development. In addition, rapid EV clearance and insufficient retention at target tissues may limit therapeutic efficacy, highlighting the need for tissue-specific targeting and sustained delivery. Biomaterial-assisted approaches, such as hydrogels and injectable scaffolds, may improve local EV retention and controlled release.

Future studies should prioritise scalable GMP-compliant production, standardised quality control and mechanism-based potency assays, and well-designed randomised, multicentre clinical trials with long-term follow-up. Next-generation strategies integrating MSC engineering, cargo modification and EV surface, biomaterials and precision medicine may further enhance EV targeting, stability and therapeutic activity. These advances could help overcome current translational barriers and accelerate the clinical development of MSC-EV therapies for musculoskeletal disorders.

## 7. Strategies to Enhance MSC-EV Therapeutic Efficacy in Musculoskeletal Disorders

Despite the promising therapeutic potential of MSC-EVs in cartilage repair, OA, RA and IVDD, their clinical translation remains limited by challenges related to production efficiency, cargo consistency, scalability and targeted delivery. Therefore, optimisation of MSC-EV production has become a critical focus for enhancing therapeutic efficacy in musculoskeletal disorders. Various engineering strategies have been developed to enhance the therapeutic efficacy of MSC-EVs, including three-dimensional (3D) culture, MSC priming, next-generation producer cell engineering, precision EV engineering, MSC-EV-inspired synthetic vesicles, biomaterial-assisted MSC-EV delivery and personalised MSC-EV therapy.

### 7.1. 3D Culture

Conventional two-dimensional (2D) monolayer culture fails to recapitulate the native physicochemical microenvironment of MSCs, often leading to suboptimal yield and therapeutic potency of MSC-EVs. In contrast, three-dimensional (3D) culture, particularly in the form of spheroids, has emerged as a powerful biomanufacturing strategy to enhance both the quantity and quality of MSC-EVs. Spheroid formation imposes a unique combination of mild hypoxia, enhanced cell-cell and cell-matrix interactions, and mechanical compaction, which remodels MSC metabolic activity and reprograms gene expression profiles. Compared with 2D culture, 3D spheroids significantly increase EV production per cell, enrich EV cargo with pro-angiogenic factors including VEGF and FGF-2, anti-inflammatory factors including IL-10 and TGF-β, and immunomodulatory microRNAs including miR-21 and miR-146a. Functionally, MSC-EVs derived from 3D spheroid cultures enhance therapeutic efficacy in preclinical models, including improved wound healing, attenuation of fibrosis, suppression of neuroinflammatory responses and protection against ischemic injury [91]. Spheroid-cultured BMSCs produce more exosomes with enriched bioactive cargo than BMSCs cultured under conventional 2D conditions. These Sph-Exos enhance NPC proliferation and ECM remodelling through miR-148a-3p- and miR-152-3p-mediated activation of the NF-κB/FOXO3 signalling pathway, thereby reducing inflammation and matrix degradation [92]. Thus, transitioning MSC expansion from 2D to spheroid-based 3D systems represents a scalable and biologically relevant approach to produce next-generation, off-the-shelf MSC-EV therapeutics with enhanced clinical potential. Exosome yield from a hollow-fibre bioreactor-based 3D culture is 7.5-fold higher than that from a 2D culture. Both 2D- and 3D-derived exosomes improve cartilage repair, but 3D exosomes demonstrate enhanced therapeutic efficacy [93]. 3D-cultured MSC-derived EVs exhibit higher VEGF expression and are better than EVs derived from 2D monolayer MSC cultures (2D-EVs) in enhancing migration and proliferation of MSCs and inflammatory chondrocytes while more effectively suppressing the expression of cartilage-degrading factors [94].

### 7.2. MSC Priming

MSC priming has emerged as an effective strategy to enhance the therapeutic efficacy of MSC-EVs. Exposing parent MSCs to defined physicochemical or biological stimuli, including inflammatory cytokines (TNF-α, IL-1β), growth factors or pharmacological agents, can significantly improve the yield, molecular cargo and functional properties of the resulting EVs. Priming effectively preconditions MSCs to adopt a more potent paracrine phenotype, which enriches EVs with pro-regenerative microRNAs, anti-inflammatory proteins and pro-angiogenic factors. Furthermore, priming can influence EV size distribution, surface receptor expression and cellular uptake efficiency. Compared to naïve MSC-EVs, primed MSC-EVs exhibit enhanced bioactivity in preclinical models of ischemic injury, acute inflammation and fibrotic disease. Consequently, priming has become a mainstream biomanufacturing step for producing next-generation, off-the-shelf MSC-EV therapeutics with enhanced and targeted clinical potential.

Curcumin-primed ADSC-EVs provide enhanced cartilage protection compared with free curcumin or unprimed small EVs. In vitro, these primed EVs more effectively reduce oxidative stress and apoptosis in stressed chondrocytes. In an OA mouse model, biweekly administration of curcumin-primed EVs significantly alleviates cartilage damage by attenuating oxidative stress and cell death, highlighting their potential as a promising therapeutic strategy for cartilage repair in OA [95]. Curcumin-primed BMSC-EVs improve intervertebral disc cartilage integrity and enhance chondrogenesis while reducing oxidative stress by restoring antioxidant capacity through PI3K/AKT/mTOR signalling and apoptosis-related proteins in IVDD [96]. Quercetin-preconditioned MSC-EVs synergistically enhance chondroprotective and anti-inflammatory effects, representing a novel OA treatment approach that integrates herbal bioactives with exosome-based delivery. Mechanistically, these MSC-EVs mitigate cartilage degradation and chondrocyte apoptosis, supporting their potential as an alternative to MSC-based therapies for OA [97]. *Artemisia argyi* (AA) possesses antioxidant, anti-inflammatory and anti-ageing properties that enhance stem cell function and improve cellular stability. AA-enhanced WJSC-derived exosomes enhance the proliferation, anti-inflammatory and antioxidant capacities of C28/I2 chondrocytes under oxidative stress conditions [98]. Nicotinamide mononucleotide (NMN)-primed infrapatellar fat pad MSC-EVs exert synergistic anti-inflammatory and cartilage-protective effects by activating MERTK signalling pathway in knee OA [99]. TNF-α preconditioning significantly increases exosome secretion from infrapatellar fat pad-derived MSCs (IPFP-MSCs) by activating the PI3K/AKT signalling pathway to upregulate ATG16L1 and enhance autophagy-associated exosome release. In an OA mouse model, intra-articular injection of TNF-α-preconditioned IPFP-MSC-derived exosomes demonstrate enhanced efficacy in alleviating joint pathology. This effect is associated with increased LRP1 expression, which contributes to enhanced chondroprotection and cartilage preservation [100]. IFN-γ preconditioning enhances the capacity and potency of the MSC secretome to stimulate cell migration and further strengthens its anti-inflammatory properties through secreted factors and EV-miRNAs [101]. Preconditioning MSCs with a pro-inflammatory cytokine cocktail of IL-1β, TNF-α and IL-17 promotes M1-to-M2 macrophage polarisation. This strategy improves the therapeutic efficacy of the MSC secretome for applications in inflammatory diseases [102].

TGF-β1-stimulated BMSC-EVs have been shown to inhibit pathological calcification while promoting balanced subchondral bone remodelling, thereby addressing both cartilage and bone pathology [86]. IL-1β-induced exosomes exhibit enhanced capacity to promote chondrocyte function, ECM production and macrophage polarisation. When encapsulated within hyaluronic acid hydrogel microspheres, these EVs achieve sustained release, which prolongs modulation of the inflammatory microenvironment and improves cartilage regeneration [103]. In addition, lipopolysaccharide (LPS) modulates the molecular composition of secreted EVs, enriching them with anti-inflammatory and regenerative factors [104]. Quercetin treatment significantly enhances exosome production and delivery efficiency, enriching EVs with antioxidant proteins such as SOD1, which suppresses NLRP3 inflammasome activation in NPCs [105]. Selenomethionine (Se-Met) promotes EV biogenesis and increases miR-125a-5p content, thereby enhancing anti-senescence effects in IVDD [106]. Parathyroid hormone (PTH)-preconditioned EVs effectively attenuate IVDD through modulation of the ZDHHC5-YBX1-ZBP1 signalling axis [107]. Conditioned medium (CM) from senescent nucleus pulposus cells (NPCs) has been used to precondition MSCs, generating “domesticated” extracellular vesicles (D-EVs) with enhanced targeting and anti-senescence properties. D-EVs selectively target senescent NPCs through the CXCL10-CXCR3 axis and deliver GPX4 protein to recipient cells, thereby suppressing ferroptosis and alleviating cellular senescence. Furthermore, incorporation of D-EVs into a ROS-responsive hydrogel enables sustained release, further enhancing their therapeutic efficacy in inhibiting NPC ferroptosis and reversing senescence-associated metabolic dysfunction [108].

Among the various priming strategies, hypoxic priming has attracted considerable attention because it closely mimics the physiological microenvironment experienced by stem cells in vivo. Exposure to low oxygen levels activates hypoxia-inducible factors (HIFs), which regulate genes involved in metabolism, angiogenesis, cell survival, immunomodulation, and EV biogenesis, ultimately improving the regenerative potential of stem cells.

Under low oxygen conditions, MSCs exhibit altered gene expression and metabolic activity, leading to increased secretion of bioactive factors such as growth factors, cytokines and extracellular vesicles. These hypoxia-induced changes can improve key therapeutic functions of MSC-EVs, including angiogenesis, immunomodulation, anti-apoptotic signalling and tissue regeneration. Consequently, hypoxia has emerged as a promising strategy to potentiate their efficacy in regenerative medicine and cell-free therapies. Hypoxic MSC-EVs exhibit superior therapeutic efficacy compared with normoxic EVs in promoting cartilage repair and reducing joint inflammation in OA models. In vitro, hypoxic MSC-EVs enhance chondrocyte proliferation, migration and ECM deposition while suppressing senescence, inflammation, matrix degradation, apoptosis and pro-inflammatory macrophage activity. These enhanced regenerative effects are associated with hypoxia-induced alterations in EV size and cargo composition, including the enrichment of specific proteins and miRNAs that coordinately regulate multiple repair pathways [15].

Mechanistically, several hypoxia-enriched miRNAs contribute to the improved therapeutic outcomes. Hypoxia-conditioned MSC-EVs promote chondrocyte proliferation and migration and suppress apoptosis through the miR-216a-5p/JAK2/STAT3 axis [109]. In addition, hypoxic MSC-EVs activate autophagy to facilitate cartilage regeneration, primarily via EV-enriched miR-122-5p, which targets DUSP2 and modulates ERK1/2 and p38 phosphorylation pathways [110]. Furthermore, hypoxic MSC-EVs contain elevated levels of hsa-miR-181c-5p and hsa-miR-18a-3p, which are predicted to promote cartilage repair through regulation of the MAPK and JAK/STAT signalling pathways, respectively [111]. Hypoxia preconditioning also generates EVs enriched in BNIP3, which activate the BNIP3/ANXA2/TFEB signalling axis to improve mitochondrial homeostasis and attenuate IVDD progression [112]. These findings highlight hypoxic MSC-EVs as a promising therapeutic strategy for the treatment of musculoskeletal disorders.

These results indicate that MSC-EV preconditioning represents a promising and scalable approach to optimise cell-free therapies, offering improved efficacy, safety and translational potential compared with untreated EVs. Primed MSC-EVs exhibit not only enhanced therapeutic efficacy in reducing inflammation, promoting tissue repair and improving overall joint outcomes, but also more consistent size distribution, cargo profiles and batch-to-batch reproducibility. These features directly address current limitations of naïve MSC-EVs, including variable therapeutic outcomes and potential off-target effects. Furthermore, preconditioning can be readily integrated into existing GMP workflows, making it a practical and commercially viable strategy for large-scale EV production. As such, primed MSC-EVs are emerging as a next-generation, off-the-shelf biologic platform with improved clinical readiness for indications in musculoskeletal disorders.

### 7.3. Next-Generation Producer Cell Engineering

Engineering MSC producer cells has emerged as a key strategy to overcome the limitations of conventional MSC-EV production, including donor-to-donor heterogeneity, batch-to-batch variability, limited expansion capacity and inconsistent therapeutic potency [113]. Advances in genome engineering, synthetic biology and stem cell technologies have enabled the development of next-generation producer cells capable of generating EVs with enhanced yield, reproducibility and therapeutic efficacy. CRISPR/Cas-mediated genome editing enables precise manipulation of genes involved in EV biogenesis, cargo sorting and therapeutic function. Editing regulators of exosome secretion or introducing therapeutic genes can increase EV production and enrich EVs with bioactive proteins, microRNAs or other therapeutic molecules. CRISPR technology also facilitates the generation of safer producer cells by eliminating undesirable genes or reducing the expression of immunogenic molecules.

Synthetic biology provides a versatile platform for programming MSCs with customised genetic circuits that enable the controlled production of therapeutic EVs. Engineered MSCs can be designed to selectively package specific miRNAs, proteins, cytokines or targeting peptides into EVs, generating “designer EVs” with enhanced cargo loading, tissue specificity and therapeutic potency [114]. MSCs overexpressing therapeutic miRNAs produce EVs enriched with regenerative and immunomodulatory cargo, resulting in improved delivery efficiency and more effective modulation of disease-associated signalling pathways. Consequently, these engineered EVs exhibit enhanced chondroprotective, cartilage-regenerative, anti-inflammatory and immunomodulatory effects, highlighting the potential of synthetic biology to overcome the limitations of native MSC-EVs and improve their clinical efficacy.

Engineered EVs can be enriched with therapeutic miRNAs to further improve their targeting capacity, bioactivity and regenerative as well as immunomodulatory functions. miR-92a-3p is enriched in MSC-derived chondrogenic exosomes but reduced in exosomes derived from OA chondrocytes. Exosomes from miR-92a-3p-overexpressing MSCs promote chondrocyte proliferation and cartilage matrix synthesis by inhibiting WNT5A, whereas inhibition of miR-92a-3p impairs chondrogenesis. In vivo, these exosomes effectively suppress cartilage degradation in OA models [115]. EVs engineered with miR-455-3p derived from 3D-cultured ADSCs significantly enhance hyaline cartilage regeneration through modulation of the miR-455-3p/PAK2/Smad2/3 signalling axis. These engineered EVs promote chondrocyte proliferation, cartilage-specific gene expression, and ECM deposition, resulting in superior cartilage repair outcomes [116]. Elevated VEGFA levels have been observed in the cartilage and synovial fluid of OA patients. miR-140 exerts protective effects by targeting VEGFA in OA. In a rat OA model, intra-articular injection of exosomes derived from miR-140-overexpressing human UC-MSCs significantly promotes cartilage regeneration and subchondral bone remodelling, outperforming control exosomes. These findings highlight modulation of the miR-140/VEGFA axis via engineered exosomes as a promising therapeutic strategy for OA [117]. MSC-EVs engineered with miR-142a-3p suppress ferroptosis and reactive ROS by directly targeting the GSK3β/Nrf2/SLC7A11 axis, protecting against cartilage matrix degradation and significantly slowing OA progression in a murine model [118]. miR-4450 is upregulated in IDD patients and TNF-α-treated NPCs. Exosome-delivered antagomiR-4450 protects NPCs in vitro and attenuates IDD progression and gait abnormalities in vivo by targeting ZNF121 [119]. EVs derived from miR-146a-overexpressing MSCs demonstrate potent immunomodulatory effects by reducing pro-inflammatory cytokines including IFN-γ, IL-17 and TNF-α while increasing anti-inflammatory cytokines such as IL-10 and IL-4, further contributing to joint protection [120]. miR-7704-enriched EVs improve functional outcomes while preserving cartilage structure through downregulation of MMP13, highlighting their chondroprotective potential [121]. In addition, miR-27b-3p-overexpressing MSC-EVs conjugated with a chondrocyte-targeting peptide (CTP) exhibit enhanced cartilage-specific targeting and significantly ameliorate OA pathology [122]. Overexpression of apoptosis linked gene-2 interacting protein X (ALIX) significantly increases MSC-sEV yield [123].

Besides miRNAs, engineered MSC-EVs with tsRNAs, lncRNAs and antisense RNAs also enhance the therapeutic efficacy. tsRNA-12391-modified ADSC-EVs promote mitophagy by binding to ATAD3A to prevent PINK1 degradation, leading to its accumulation, thereby alleviating cartilage degeneration [124]. LncRNA MALAT-1-overexpressing MSC-EVs promote chondrocyte proliferation, reduce inflammation and cartilage degeneration and enhance repair capacity by decreasing MMP-13, IL-6 and Caspase-3 expression, suggesting that MALAT-1-enriched hMSC-EVs represent a promising therapeutic approach for OA [125]. EVs derived from MSCs overexpressing KLF3-AS1 further protect chondrocytes by acting as a competing endogenous RNA (ceRNA) that sponges miR-206, thereby upregulating GIT1 to promote proliferation and inhibit apoptosis. These effects are reversed by miR-206 overexpression or GIT1 knockdown, highlighting the KLF3-AS1/miR-206/GIT1 axis as a key regulatory mechanism [126].

To address the challenges associated with primary MSCs, stable producer cell lines have been established through immortalization [17,127]. These cell lines retain robust EV secretion while exhibiting prolonged proliferative capacity and reduced donor variability, making them attractive platforms for large-scale, cost-effective manufacturing. Stable producer cells also facilitate process standardisation, quality control and compliance with GMP requirements. Among emerging platforms, iPSC-derived MSC master cell banks offer a renewable and highly standardised source of producer cells [128]. Because iPSCs possess unlimited self-renewal capacity, a single well-characterised master cell bank can generate large numbers of genetically uniform MSCs for long-term EV production. Furthermore, patient-specific or HLA-engineered iPSC lines may provide opportunities for personalised or universal MSC-EV therapies. Combined with genome editing and chemically defined culture systems, iPSC-derived MSCs enable the scalable production of clinical-grade EVs with improved consistency, reduced batch variation and enhanced safety [3].

Advances in producer cell engineering are shifting MSC-EV development from conventional EV production toward the generation of more defined, programmable and therapeutically optimised EV products. By simultaneously controlling EV biogenesis, cargo loading, membrane composition, targeting properties and the cellular microenvironment, engineered MSCs offer opportunities to improve EV yield, functional potency, specificity and batch-to-batch consistency. The integration of stable producer cell platforms, multi-omics-guided engineering and scalable bioreactor manufacturing may further facilitate the development of standardised MSC-EV products with improved translational potential. Nevertheless, challenges related to engineering stability, manufacturing scalability, product heterogeneity, safety and regulatory standardisation will need to be addressed before these next-generation producer cell platforms can be broadly applied in clinical settings.

### 7.4. Precision EV Engineering

MSC-EVs loaded with specific miRNAs, siRNAs, drugs or liposomes represent an additional strategy to enhance therapeutic efficacy. Knee osteoarthritis (KOA) is a prevalent orthopaedic disorder with multifactorial aetiology, including genetic and biomechanical factors. Engineered EVs loaded with miR-29a exhibit anti-inflammatory effects and preserve ECM stability, thereby protecting articular cartilage and slowing KOA progression [129]. EVs loaded with MMP13 siRNA and functionalized with IGF-1 exhibit enhanced cartilage targeting and retention, leading to more effective and longer suppression of matrix degradation and promotion of cartilage regeneration [130]. MSC-derived EVs loaded with siMDM2 and functionalized with the cartilage-targeting peptide WYRGRL demonstrate improved chondrocyte uptake, deeper cartilage penetration and prolonged joint retention. This multifunctional design enhances anti-ageing effects by selectively eliminating senescent chondrocytes and maintaining matrix homeostasis [131]. EVs loaded with the anti-inflammatory drug celecoxib improve modulation of the inflammatory microenvironment and promote cartilage repair, particularly when derived from 3D-cultured MSCs, which further enhances EV potency [132]. A hybrid nanoparticle engineered with transforming growth factor-beta1 (TGF-β1)-overexpressing EVs and cartilage-targeted anti-inflammatory liposomes represents a promising therapeutic strategy for OA. This platform enables precise targeting and sustains therapeutic action, leading to robust anti-inflammatory and chondroprotective effects within osteoarthritic lesions [133].

Native MSC-EVs possess intrinsic homing and cell-interaction properties, but their biodistribution after systemic administration is generally not sufficiently selective. Membrane engineering has therefore emerged as an important strategy to enhance EV targeting, cellular uptake, circulation stability and tissue accumulation while minimising nonspecific interactions and off-target effects. Unlike cargo engineering, which primarily modifies the molecular contents of EVs, membrane engineering focuses on the EV surface to promote tissue-specific delivery and minimise off-target effects. Current approaches can be broadly categorised into genetic engineering of EV-producing MSCs, direct chemical modification of isolated EVs and hybrid or physical membrane engineering.

Genetic engineering enables the display of targeting ligands on EV surfaces by fusing peptides, proteins, antibodies, or nanobodies to EV-associated membrane proteins, including Lamp2b, CD9, CD63, CD81, and PDGFR, or to membrane-anchoring motifs such as glycosylphosphatidylinositol (GPI). Lamp2b is widely used as a membrane scaffold to display targeting peptides or proteins on the outer EV surface. Engineering EV-associated scaffold proteins, such as Lamp2b, CD9, CD63, CD81, and PDGFR, enables the incorporation of targeting ligands, antibody fragments or functional membrane proteins into the EV membrane, thereby enhancing tissue-specific targeting and the efficiency of EV delivery. This approach involves genetically modifying parental MSCs to express the desired fusion proteins, which are subsequently incorporated into the membrane of secreted EVs and presented on their surface [134]. CAP-LAMP2b engineering enhances hUCMSC-EV uptake by chondrocytes, while liposome fusion enables the delivery of CRISPR-Cas9 ADAMTS4 gRNA. CAP-LAMP2b-ADAMTS4 EVs suppress IL-1β-induced ADAMTS4 expression and inflammation, restore aggrecan levels and preserve cartilage matrix integrity, demonstrating enhanced therapeutic efficacy [135].

In contrast, chemical approaches directly functionalise isolated EV membranes through PEGylation, covalent conjugation, lipid insertion or click chemistry, offering greater flexibility and avoiding permanent genetic modification of the producer cells. PEGylation can further improve EV stability and circulation by reducing nonspecific protein adsorption and cellular interactions, while incorporating targeting ligands can preserve or enhance receptor-specific recognition [136]. Lipid-PEG conjugates and streptavidin-based systems also provide modular platforms for attaching targeting molecules to the EV surface [137]. In addition, hybrid membrane engineering, including EV–liposome membrane fusion, integrates the intrinsic biological functionality of EV membranes with the tunable physicochemical properties of synthetic liposomes [138,139]. These approaches provide complementary means of tailoring MSC-EV surface properties and represent an important avenue for developing more precise and effective EV-based therapeutics. However, potential alterations in membrane integrity, endogenous membrane proteins, EV biogenesis, surface charge and ligand orientation remain important considerations for optimising engineered MSC-EVs and facilitating their clinical translation.

### 7.5. MSC-EV-Inspired Synthetic Vesicles

Native MSC-EVs have limited capacity for precise and controllable loading of defined therapeutic cargos. MSC-EV-inspired synthetic vesicles have therefore emerged as a promising next-generation platform to overcome several limitations of native MSC-EVs, including relatively low production yields, donor-to-donor heterogeneity, limited cargo loading capacity, and challenges associated with large-scale manufacturing [140]. Depending on their design and method of production, these vesicles can be broadly classified into EV-mimetic nanovesicles, cell membrane-derived nanovesicles, synthetic liposomes, and hybrid vesicles.

EV-mimetic nanovesicles can be generated by mechanically disrupting MSCs through serial extrusion or related microfluidic approaches, producing nanoscale vesicles that retain selected MSC membrane proteins and associated biological activities while achieving substantially higher yields than naturally secreted EVs [141,142]. Similarly, cell membrane-derived nanovesicles preserve key membrane proteins and receptors from MSCs, thereby retaining intrinsic biocompatibility and cell-recognition or targeting properties while providing greater flexibility in vesicle production and cargo incorporation [141,143].

Synthetic liposomes provide a highly controllable and versatile platform for encapsulating therapeutic proteins, nucleic acids and small-molecule drugs. However, they generally lack the complex endogenous membrane proteins and associated biological functions of native EVs that contribute to cellular recognition, targeting and intercellular communication [144]. To overcome these limitations, hybrid vesicles generated by fusing MSC-EVs with synthetic liposomes or other nanomaterials seek to combine the intrinsic targeting capacity, low immunogenicity and biological activity of EVs with the high cargo loading capacity, improved stability, tunable physicochemical properties and manufacturing flexibility of synthetic nanocarriers. Such hybrid systems can facilitate the incorporation of diverse therapeutic cargos while potentially enhancing vesicle stability, circulation and tissue-specific delivery [145].

### 7.6. Biomaterial-Assisted Delivery of MSC-EVs

Biomaterial-assisted delivery systems have emerged as an effective strategy to overcome the rapid clearance, limited retention and poor local bioavailability of MSC-EVs following administration. By providing structural support and controlled release, biomaterials protect EVs from degradation, prolong their residence time at the injury site and enhance their therapeutic efficacy [146,147]. Hydrogels are among the most widely investigated carriers for EV delivery owing to their high water content, injectability and biocompatibility. Their incorporation of EVs enables localised and sustained EV release, prolongs EV bioavailability and provides a favourable, cartilage-mimetic microenvironment that supports cell survival, extracellular matrix (ECM) deposition, tissue integration, and regeneration [148,149]. Pluronic F127 hydrogel encapsulating SCNN1B-engineered adipose MSC-EVs significantly improves tracheal cartilage regeneration through activation of PERK/ATF4 signalling in rat models compared with control EVs [150]. In addition, biomimetic hydrogels incorporating exosomes have been shown to promote MSC recruitment, migration and chondrogenesis while simultaneously enhancing ECM synthesis and cartilage integration at injury sites [151]. These combinatorial strategies improve mechanical stability, prolong therapeutic efficacy and better recapitulate the native cartilage niche, thereby overcoming challenges associated with rapid EV clearance and insufficient retention in vivo.

Biodegradable scaffolds, including natural and synthetic polymer-based constructs, further improve EV localization by providing mechanical support and promoting cell adhesion, migration and tissue integration [152,153,154]. These scaffolds can be engineered to achieve spatiotemporal EV release, thereby enhancing chondrogenesis, angiogenesis and osteochondral repair. More recently, microneedle-based delivery systems have attracted increasing attention as a minimally invasive platform for localised EV administration. Microneedles facilitate efficient penetration into inflamed joints, enabling sustained EV delivery while minimising systemic exposure and improving patient compliance [155]. In addition, emerging biomaterial platforms, such as 3D-printed scaffolds, adhesive hydrogels and stimulus-responsive biomaterials, offer programmable release kinetics and responsive EV delivery under disease-specific conditions. These biomaterial-based approaches significantly enhance the stability, retention and therapeutic efficacy of MSC-EVs, representing promising strategies to improve cartilage regeneration and accelerate the clinical translation of MSC-EV-based therapies.

### 7.7. Personalised MSC-EV Therapy

Personalised MSC-EV therapy represents an emerging strategy in regenerative medicine that aims to tailor EV-based interventions to the biological characteristics of individual patients and disease states [113]. Although MSC-EVs possess intrinsic immunomodulatory, anti-inflammatory and tissue-reparative activities, their composition and therapeutic potency can vary substantially depending on the MSC source, donor characteristics, tissue origin, culture conditions and disease microenvironment. Such heterogeneity may contribute to differences in EV biodistribution, cargo composition and therapeutic efficacy among patients. Therefore, integrating patient-specific biological information with optimised MSC sources and EV engineering strategies may enable the development of more precise and effective EV therapies.

One approach to personalization is the selection of MSCs based on donor- or tissue-specific characteristics associated with therapeutic potency [156]. Bone marrow-, adipose tissue-, umbilical cord-, and other tissue-derived MSCs produce EVs with distinct molecular cargo profiles and biological activities. In addition, preconditioning of MSCs with hypoxia, inflammatory cytokines, growth factors or pharmacological agents can modify EV cargo and enhance specific therapeutic functions. This provides an opportunity to generate disease-tailored MSC-EVs with enhanced anti-inflammatory, immunomodulatory, angiogenic or tissue-regenerative properties. EV engineering further expands the potential for personalised therapy. Surface engineering can incorporate targeting peptides, ligands, antibodies or membrane proteins to improve delivery to disease-relevant tissues or cell populations, whereas cargo engineering can enrich EVs with therapeutic miRNAs, mRNAs, proteins or gene-editing components. Combining these approaches with patient-specific disease biomarkers could enable the design of MSC-EVs that selectively target pathological tissues and modulate disease-associated molecular pathways while minimising off-target effects.

Personalised MSC-EV therapy may be particularly valuable for musculoskeletal diseases, as cartilage disorders are characterised by substantial interpatient heterogeneity in inflammatory status, extracellular matrix remodelling, disease stage and cellular responses to therapy [157]. Patient-derived molecular profiles, including inflammatory cytokines, matrix-degrading enzymes and transcriptomic or proteomic signatures, could potentially be used to identify the most appropriate EV source, therapeutic cargo, targeting strategy and dosing regimen. Patients with predominantly inflammatory osteoarthritis may benefit from MSC-EVs enriched in immunomodulatory factors, whereas patients with severe matrix degradation may require EVs engineered to deliver anabolic or anti-catabolic cargos. The development of personalised MSC-EV therapy will nevertheless require robust potency assays, standardised manufacturing and reliable biomarkers for patient stratification. Integration of multi-omics profiling, single-EV analysis, artificial intelligence and high-throughput screening could facilitate the identification of EV characteristics associated with therapeutic response and support rational matching of EV products to individual disease phenotypes. Ultimately, a precision medicine framework combining patient stratification, optimised MSC-EV manufacturing, molecular engineering and individualised dosing may improve the reproducibility and clinical efficacy of MSC-EV-based therapies for cartilage repair, OA, RA and IVDD, thereby accelerating the clinical adoption of MSC-EV-based regenerative medicine.

## 8. Limitations of MSC-EV-Based Therapies

Despite encouraging preclinical evidence, several challenges remain before MSC-EVs can be broadly translated into clinical therapies for OA, RA and IVDD. A major limitation is the considerable heterogeneity of MSC-EV products arising from differences in MSC tissue source, donor characteristics, passage number, culture conditions, EV isolation methods and storage procedures. These factors can substantially influence EV yield, cargo composition and biological activity. Furthermore, the lack of standardised protocols for EV production, characterisation, dosing and potency assessment complicates comparisons between studies and the development of reproducible manufacturing processes.

Disease-specific factors may also influence the therapeutic efficacy of MSC-EVs. In OA, EV-mediated effects on cartilage homeostasis and inflammation may vary according to disease stage, joint inflammation, mechanical loading and patient characteristics. OA is a heterogeneous disease arising from diverse etiologies and risk factors, including joint trauma, obesity and metabolic dysfunction, ageing and postmenopausal changes. These distinct disease drivers may involve different pathological mechanisms and tissue responses, potentially resulting in variable responses to MSC-EV therapy. Thus, patient stratification according to disease aetiology and molecular or clinical characteristics may be important for developing personalised EV-based interventions. In RA, the systemic and complex autoimmune nature of the disease presents additional challenges, as effective therapy may require sustained modulation of multiple immune pathways rather than local suppression of inflammation alone. The potential interactions between MSC-EVs and existing disease-modifying antirheumatic drugs also warrant further investigation. In IVDD, the avascular, hypoxic, acidic and mechanically stressed microenvironment of the degenerated disc may restrict EV diffusion, retention and biological activity following administration.

Another important challenge is achieving effective biological repair in tissues exposed to disease-altered biomechanical environments. In OA and IVDD, disease progression involves changes in mechanical loading, extracellular matrix composition, tissue stiffness and tissue architecture. These pathological biomechanical conditions may impair endogenous repair and limit the ability of MSC-EVs to restore tissue integrity, even when they effectively modulate inflammation, promote cell survival or enhance extracellular matrix synthesis. Therefore, durable regeneration may require approaches that address both biological and biomechanical abnormalities, such as combining MSC-EVs with biomaterials, mechanical support or strategies that restore the extracellular matrix and tissue biomechanics.

Finally, limited clinical evidence remains a critical barrier to translation. This review does not encompass all aspects of MSC-EV biology, manufacturing, delivery and therapeutic applications, and several emerging areas remain beyond the scope of the present discussion. Most studies are based on cell or animal models that may not fully recapitulate the complexity and heterogeneity of human disease. Important questions regarding EV pharmacokinetics, biodistribution, optimal dose and administration frequency, long-term safety and potential off-target effects remain unresolved. Standardised GMP-compatible manufacturing, robust potency assays, improved delivery strategies and well-controlled clinical trials are therefore essential to establish the safety, consistency and therapeutic efficacy of MSC-EVs for musculoskeletal diseases.

## 9. Conclusions and Future Perspectives

MSC-EVs exert broad therapeutic effects across musculoskeletal disorders by regulating multiple cellular processes and signalling networks. In cartilage regeneration, they enhance stem cell recruitment, chondrocyte proliferation and migration, stimulate ECM synthesis and promote hyaline cartilage formation while suppressing inflammation, apoptosis and oxidative stress. In OA, MSC-EVs modulate key signalling pathways, including NF-κB, MAPK, Wnt/β-catenin, YAP, PI3K-Akt, ERK and AMPK, thereby preserving cartilage homeostasis, reducing catabolic activity and limiting disease progression. In RA, MSC-EVs coordinate innate and adaptive immune responses, metabolic reprogramming and tissue repair processes, contributing to the attenuation of chronic inflammation. In IVDD, MSC-EVs target apoptosis, senescence, ferroptosis, pyroptosis, ECM degradation and inflammatory cascades, improving nucleus pulposus cell survival and matrix integrity. These multi-target and cell-free mechanisms enable MSC-EVs to restore tissue homeostasis and demonstrate strong therapeutic potential for cartilage repair, OA, RA and IVDD in preclinical models.

However, the clinical translation of MSC-EVs remains constrained by several challenges, including low production efficiency, cargo heterogeneity, scalability constraints, incomplete understanding of their mechanisms of action, lack of standardised purification methods, insufficient characterisation assays and regulatory challenges. Accordingly, optimising MSC-EV production has become a key priority for improving therapeutic efficacy in musculoskeletal disorders. Future research should focus on enhancing production yield, improving targeting and retention, elucidating mechanisms of action, and establishing standardised, GMP-compliant manufacturing and quality control systems. Scalable bioprocessing strategies, such as bioreactor-based expansion and 3D culture systems, are required to improve yield and batch-to-batch consistency, while advanced purification techniques and automation can support the generation of clinical-grade EV products. In parallel, engineering approaches, including surface modification and biomaterial-based delivery systems, may enhance EV localization and prolong therapeutic activity, particularly in poorly vascularized tissues such as the intervertebral disc. Furthermore, standardised characterisation protocols and validated potency assays are essential for ensuring product reproducibility, defining critical quality attributes and facilitating regulatory approval.

Ultimately, successful clinical translation will require an integrated approach encompassing standardised manufacturing, rigorous quality control, disease-specific patient stratification, optimised delivery and well-designed clinical trials. With continued advances in EV bioprocessing, engineering, characterisation and delivery technologies, MSC-EVs hold considerable promise as a next-generation cell-free therapeutic platform for musculoskeletal disorders.

## Figures and Tables

**Table 1:** Effects and mechanisms of MSC-EVs in OA, RA and IVDD.

EV Source	Disease Model	Effects	Mechanism	References
Synovial MSC	OA	Drive chondrocyte proliferation and migration	Activate the KDM2A/E2F1/PTTG1 axis via miR-31	[32]
BMSC	OA	enhances migratory capacity and protects against cartilage degeneration	Via exosomal miR-136-5p	[33]
DPSC	OA	promote chondrocyte proliferation and migration while simultaneously reducing inflammation	Via ERK-CREB-CGRP signaling pathway	[34]
WJMSC	OA	Enhance proliferation and chondrogenesis	Exosomal miRNA activates calcium signaling, NOTCH and ECM-receptor interaction pathways	[35]
Synovial MSC	OA	MSC-EVs enhanced chondrocyte and synovial MSC growth and migration	Upregulate CXCL5 and CXCL6	[36]
ADSC	OA	Mitigate cartilage degeneration	Suppress multiple inflammatory cytokines and upregulating FGF 18	[37]
BMSC	OA	Enhance chondrocyte proliferation, reduce apoptosis and suppress inflammatory cytokines including TNF-α, IL-6 and IL-1β	Inhibit NF-κB p65 phosphorylation	[38]
SMSC	OA	Suppress apoptosis and ameliorate cartilage damage	Deliver miR-26a-5p into chondrocytes to inhibit PTEN	[39]
hMSC	OA	Suppress chondrocyte apoptosis, facilitate cartilage repair	Regulate KLF3-AS1/miR-206/GIT1 axis, suppress IL-1β-induced inflammatory responses	[40]
BMSC	OA	Inhibits pyroptosis	Target HDAC3 and downregulating the STAT1/NF-κB signaling pathway	[41]
BMSC	OA	Suppresses inflammation, alleviate Osteoarthritis	Modulate the miR-485-5p/FSP1 axis	[42]
ADSC	OA	Reduces inflammation and cell death	Activate the PINK1/Parkin pathway to promote mitochondrial autophagy and restore mitochondrial homeostasis	[43]
UC-MSC	OA	Supports chondrocyte survival	Reduce oxidative stress and cellular senescence while enhancing mitochondrial function	[44,45]
UC-MSC	OA	Promoted cartilage regeneration	Downregulate the expression of MMP-13 and ADAMTS-5, upregulated collagen II expression, attenuate inflammation	[46]
ESC-MSC	OA	Reduce aggrecan degradation, maintain ECM stability	Preserve collagen II synthesis, markedly inhibit ADAMTS5 expression	[47]
BMSC	OA	Alleviate chondrocyte injury, reduce the transcription of pro-inflammatory cytokines	Target DDX20 and suppress NF-κB activation	[48]
BMSC	OA	Protects against chondrocyte apoptosis and ECM degradation, preserve cartilage structure	Suppress the SDC1/Wnt/β-catenin pathway	[49]
BMSC	OA	Inhibit the OA progression	Downregulate catabolic enzymes while promoting ECM synthesis	[50]
DPSC	OA	Promote cartilage regeneration and relieve pain	Inhibit the ERK-CREB-CGRP pathway	[34]
SMSC	OA	Enhancing chondrocyte proliferation and migration, repair cartilage	Exosomal Wnt5a and Wnt5b activate YAP signaling through the non-canonical Wnt pathway	[51]
UC-MSC	OA	Prevent OA progression	Suppress the secretion of pro-inflammatory factors while enhancing the expression of cartilage-specific ECM markers	[52]
SMSC	OA	Attenuate chondrocyte inflammation	miR-129-5p targets HMGB1	[53]
BMSC	OA	Enhance autophagy and cellular stress resistance	lncRNA NEAT1 activates the miR-122-5p/Sesn2/NRF2 signaling axis	[54]
BMSC	OA	Alleviates OA progression	circYAP1 regulates the miR-21/TLR7 pathway	[55]
MSC	OA	Prevent the development of OA	circHIPK3/miR-124-3p/MYH9 axis	[56]
UC-MSC	OA	Promote a switch toward the anti-inflammatory M2	Modulates STAT1 signaling	[57,58]
BMSC	OA	Ameliorate cartilage damage	Repress M1 polarization while promoting M2 polarization via miR-135b/MAPK6 signaling	[59]
UC-MSC	OA	Support chondrocyte survival and ECM synthesis	Modulate PI3K-Akt pathway through specific miRNAs	[58]
ADSC	OA	Suppress chondrocyte hypertrophy and inflammatory response	Exosomal miR-574-3p regulates the CRIM1/BMP signaling axis	[60]
AF-MSC	OA	Exert anti-inflammatory effects against IL-1β and TNF-α	Suppress ERK phosphorylation in the MAPK pathway	[61]
BMSC	OA	Attenuate IL-1β-induced chondrocyte injury	Through AKT/ERK/AMPK signaling	[62]
BMSC	OA	Promote cartilage regeneration	Through autotaxin-YAP signaling	[63]
Gingiva-MSC	RA	Restore the balance between Th17 and Treg	Modulate the IKKβ/NF-κB signaling pathway via miR-148a-3p	[11]
ESC-MSC	RA	Reduce synovial inflammation and attenuate disease progression	Promote the transition of M1 toward M2	[64]
ADSC	RA	Alleviates rheumatoid arthritis	Modulate macrophage polarization	[65]
Immortalized MSC	RA	Reinforce the immunosuppression	Enhance TGF-β1 production and promote M2 polarization, suppress pro-inflammatory cytokines	[66]
BMSC	RA	Alleviate RA symptoms	Likely through the delivery of proteins associated with cell adhesion, tissue remodeling and regenerative processes	[67]
BMSC	IVDD	Counteract ferroptosis	Activate antioxidant and cytoprotective signaling pathways, particularly the p62/KEAP1/NRF2 axis	[68]
BMSC	IVDD	Enhanced NPC proliferation and improved ECM synthesis	Inhibits nucleus pulposus cell ferroptosis to relieve IVDD through circ_0072464/miR-431/NRF2	[69]
BMSC	IVDD	Reduce pyroptosis-mediated NPC injury, inflammasome activation	Suppress NLRP3 signaling through delivery of miR-410	[70]
BMSC	IVDD	Attenuate TNF-α-induced NPC apoptosis	Target PTEN to activate the PI3K/Akt signaling pathway	[71]
BMSC	IVDD	Inhibits apoptosis, ECM breakdown and fibrosis	Target RASSF5 via exosomal miR-532-5p	[72]
BMSC	IVDD	Suppress apoptosis, attenuate ECM degradation	Target EZH2 to relieve transcriptional repression of FOXO3 via miR-217	[73]
BMSC	IVDD	Alleviate oxidative stress-induced mitochondrial dysfunction	Preserve mitochondrial membrane potential, reduce excessive ROS accumulation and limit apoptosis	[74]
BMSC	IVDD	Alleviate NPC degeneration	Through the miR-145a-5p/USP31/HIF-1alpha Signaling Pathway	[75]
iMSC	IVDD	Modulate senescent nucleus pulposus cells	Partly through miR-100-5p-mediated suppression of mTORC1 signaling and regulation of glycolytic metabolic reprogramming	[76]
BMSC	IVDD	Slow IVDD progression	Activate key inflammatory mediators and inhibit NLRP3 inflammasome signaling in NPCs	[77]
BMSC	IVDD	Inhibit NPC apoptosis	miR-125b-5p/TRAF6/NF-kappaB pathway axis	[78]

**Table 2:** Summary of clinical trials evaluating MSC-EVs for osteoarthritis (OA) and cartilage repair.

ClinicalTrials.gov ID	EV Source	Disease	Patients	Administration	Phase	Follow-Up Period	Primary Aims
NCT06466850	Allogeneic MSCs	Knee OA	*n* = 20	Intra-articular	Phase 1	1, 3 and 6 months	Assess the efficacy
NCT05060107	Allogeneic UC-MSCs	Knee OA	*n* = 10	Intra-articular	Phase 1	12 months	Assess safety and efficacy
NCT06431152	Allogeneic UC-MSCs	Knee OA	*n* = 12	Intra-articular	Phase 1	12 months	Evaluate safety, tolerability and potential immunomodulatory/regenerative effects in joint tissues
NCT05261360	Autologous SF-MSCs	Meniscus OA	*n* = 10	Intra-articular	Phase 2	12 months	Assess clinical efficacy
NCT06937528	EVs	Knee OA	*n* = 50	Intra-articular	Phase 1	12 months	Evaluate the safety and efficacy
NCT04223622	ADSCs	OA or articular regeneration	*n* = 24	Intra-articular	Phase 1	3 years	Assess the efficacy

## Data Availability

No new data were created or analyzed in this study. Data sharing is not applicable to this article.

## Supplementary Materials

The following supporting information can be downloaded at: https://www.mdpi.com/article/10.3390/ijms27188208/s1.

## Abbreviations

2D	Two-dimensional
3D	Three-dimensional
AA	*Artemisia argyi*
ADAMTS5	A disintegrin and metalloproteinase with thrombospondin motifs 5
ADSC	Adipose mesenchymal stem cells
AF-MSC	Amniotic fluid-derived MSC
AKT	Protein kinase B
ALIX	apoptosis-linked gene-2 interacting protein X
AMPK	AMP-activated protein kinase
ANXA2	Annexin A2
ATAD3A	ATPase family AAA domain-containing 3A
BMSC	Bone marrow-derived MSCs
BNIP3	BCL2 interacting protein 3
Cas	CRISPR-associated proteins
CCL5	C-C Motif Chemokine Ligand 5
ceRNA	Competing endogenous RNA
CGRP	Calcitonin gene-related peptide
CIA	Collagen-induced arthritis
circRNAs	Circular RNAs
COL2A1	Collagen type II alpha 1 chain
COX2	Cyclooxygenase-2
CREB	cAMP response element-binding protein
CRIM1	Cysteine rich transmembrane BMP regulator 1
CRISPR	Clustered Regularly Interspaced Short Palindromic Repeats
CTP	Chondrocyte-targeting peptide
CXCL5	C-X-C motif chemokine ligand 5
CXCL6	C-X-C motif chemokine ligand 6
CXCR2	C-X-C motif chemokine receptor 2
DDX20	DEAD-box helicase 20
DMARD	Disease-modifying anti-rheumatic drug
DPSC	Dental pulp stem cells
E2F1	E2F transcription factor 1
E2F2	E2F transcription factor 2
ECM	Extracellular matrix
ERK	Extracellular signal-regulated kinase
ESC-MSC	Embryonic stem cell-derived MSC
EV	Extracellular vesicle
EZH2	Enhancer of zeste 2 polycomb repressive complex 2 subunit
FGF18	Fibroblast growth factor 18
FGF2	Fibroblast growth factor 2
fMSCs	Foetal MSCs
FOXO3	Forkhead box O3
FSP1	Fibroblast-specific protein 1
GIT1	G protein-coupled receptor kinase-interacting ArfGAP 1
GLUT1	Glucose transporter 1
GMP	Good Manufacturing Practice
GMSC	Gingiva-derived MSC
GSK3β	Glycogen synthase kinase 3 beta
GVHD	Graft-versus-host disease
HIF-1α	Hypoxia-inducible factor 1 alpha
HMGB1	High mobility group box 1
IFN-γ	Interferon gamma
IKKβ	Inhibitor of nuclear factor kappa B kinase subunit beta
IL-10	Interleukin-10
IL-17	Interleukin-17
IL-1β	Interleukin-1 beta
IL-6	Interleukin-6
ILVs	Intraluminal vesicles
iMSCs	Induced pluripotent stem cell-derived MSCs
IPFP-MSC	Infrapatellar fat pad-derived MSC
ITGB1	Integrin Beta-1
IVDD	Intervertebral disc degeneration
JAK2	Janus kinase 2
JNK	c-Jun N-terminal kinase
KDM2A	Lysine demethylase 2A
KEAP1	Kelch-like ECH-associated protein 1
KLF3	Kruppel-like factor 3
KOA	Knee osteoarthritis
lncRNAs	Long non-coding RNAs
LPS	Lipopolysaccharide
LRP1	Low-density lipoprotein receptor-related protein 1
MAPK	Mitogen-activated protein kinase
MDM2	MDM2 proto-oncogene
METTL3	Methyltransferase-like 3
miRNAs	microRNAs
MISEV	Minimal Information for Studies of Extracellular Vesicles
MMP13	Matrix Metalloproteinase-13
MMP3	Matrix Metalloproteinase-3
MSC	Mesenchymal stem cell
MSC-EVs	Mesenchymal stem cell-derived extracellular vesicles
MVBs	Multivesicular bodies
MYH9	Myosin heavy chain 9
NanoFCM	Nano-flow cytometry
NEAT1	Nuclear paraspeckle assembly transcript 1
NF-κB	Nuclear factor kappa-light-chain-enhancer of activated B cells
NLRP3	NLR family pyrin domain containing 3
NOX4	NADPH Oxidase 4
NPC	Nucleus pulposus cell
NRF2	Nuclear factor erythroid 2-related factor 2
NTA	Nanoparticle tracking analysis
OA	Osteoarthritis
p38	p38 mitogen-activated protein kinase
p62	Sequestosome 1
PAK2	p21 (RAC1) activated kinase 2
PEG	Polyethylene glycol
PHLDA2	Pleckstrin homology like domain family A member 2
PI3K	Phosphoinositide 3-kinase
PINK1	PTEN induced kinase 1
PTEN	Phosphatase and tensin homologue
PTH	Parathyroid hormone
PTTG1	Pituitary tumour-transforming 1
RA	Rheumatoid arthritis
RalA	RAS like proto-oncogene A
RASSF5	Ras association domain family member 5
ROS	Reactive oxygen species
RUNX2	RUNX family transcription factor 2
SCNN1B	Sodium Channel Epithelial 1 Subunit Beta
SIRT6	Sirtuin 6
SLC7A11	Solute carrier family 7 member 11
SMAD2	SMAD family member 2
SMAD3	SMAD family member 3
SMSC	Synovial mesenchymal stem cell
SNHG7	Small nucleolar RNA host gene 7
SOX9	SRY-box transcription factor 9
STAT1	Signal transducer and activator of transcription 1
STAT3	Signal transducer and activator of transcription 3
TEM	Transmission electron microscopy
TFEB	Transcription factor EB
TGF-β	Transforming Growth Factor-beta
TLR7	Toll-like receptor 7
TMJOA	Temporomandibular joint osteoarthritis
TNF-α	Tumour Necrosis Factor-alpha
TRAF6	TNF receptor associated factor 6
TRPS	Tunable resistive pulse sensing
UC-MSC	Umbilical cord-derived MSC
USP31	Ubiquitin specific peptidase 31
VEGFA	Vascular endothelial growth factor A
WJ-MSC	Wharton’s jelly MSCs
YAP	Yes-associated protein
ZNF121	Zinc finger protein 121
