# Supplementary material for: MSC-EVs in Cartilage Regeneration and Immunomodulation: Mechanisms and Therapeutic Prospects for Osteoarthritis, Rheumatoid Arthritis and Intervertebral Disc Degeneration
Source: Int J Mol Sci. 2026 Sep 15;27(18):8208. doi: 10.3390/ijms27188208 (PMC13607499; doi:10.3390/ijms27188208)
Supplement: Supplementary file 1 [file ijms-27-08208-s001.zip › ijms-4380257-supplementary.pdf]

Figure S1

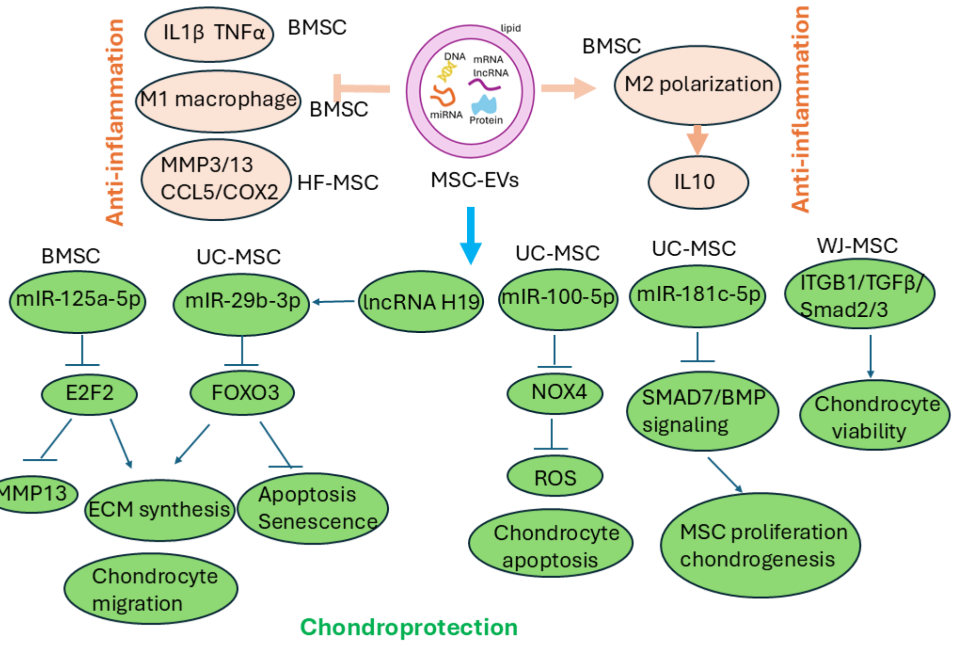

Figure S1. MSC-EVs promote cartilage repair by multiple reparative processes.

Mesenchymal stem cell-derived extracellular vesicles (MSC-EVs) promote cartilage regeneration by enhancing chondrocyte and MSC migration, proliferation, chondrogenesis and extracellular matrix (ECM) synthesis while suppressing apoptosis, senescence, oxidative stress and inflammation through multiple miRNA- and signaling pathways. They also promote M2 macrophage polarization and IL-10 secretion, thereby reducing pro-inflammatory mediators and catabolic enzymes and supporting joint tissue homeostasis.
